# LAMP3 induces apoptosis and autoantigen release in Sjögren’s syndrome patients

**DOI:** 10.1038/s41598-020-71669-5

**Published:** 2020-09-16

**Authors:** Tsutomu Tanaka, Blake M. Warner, Toshio Odani, Youngmi Ji, Ying-Qian Mo, Hiroyuki Nakamura, Shyh-Ing Jang, Hongen Yin, Drew G. Michael, Noriyuki Hirata, Futoshi Suizu, Satoko Ishigaki, Fabiola Reis Oliveira, Ana Carolina F. Motta, Alfredo Ribeiro-Silva, Eduardo M. Rocha, Tatsuya Atsumi, Masayuki Noguchi, John A. Chiorini

**Affiliations:** 1 National Institute of Dental and Craniofacial Research, National Institutes of Health, NIH 10 Center Dr., Bethesda, MD 20892 USA; 2 Division of Cancer Biology, Institute for Genetic Medicine, Hokkaido University, Sapporo, Japan; 3 Department of Clinical Medicine, Ribeirão Preto Medical School, University of Sao Paulo, Ribeirao Preto, Brazil; 4 Department of Stomatology, Public Health and Forensic Dentistry, School of Dentistry of Ribeirão Preto, University of Sao Paulo, Ribeirao Preto, Brazil; 5 Department of Pathology and Legal Medicine, Ribeirao Preto Medical School, University of Sao Paulo, Ribeirao Preto, SP Brazil; 6 Department of Ophthalmology, Otorhinolaryngology, Head and Neck Surgery, Ribeirão Preto Medical School, University of Sao Paulo, Ribeirao Preto, Brazil; 7 Department of Rheumatology, Endocrinology and Nephrology, Faculty of Medicine and Graduate School of Medicine, Hokkaido University, Sapporo, Japan

**Keywords:** Cell biology, Immunology, Molecular biology, Biomarkers

## Abstract

Primary Sjögren’s syndrome (pSS) is a complex autoimmune disease characterized by dysfunction of secretory epithelia with only palliative therapy. Patients present with a constellation of symptoms, and the diversity of symptomatic presentation has made it difficult to understand the underlying disease mechanisms. In this study, aggregation of unbiased transcriptome profiling data sets of minor salivary gland biopsies from controls and Sjögren’s syndrome patients identified increased expression of lysosome-associated membrane protein 3 (LAMP3/CD208/DC-LAMP) in a subset of Sjögren’s syndrome cases. Stratification of patients based on their clinical characteristics suggested an association between increased LAMP3 expression and the presence of serum autoantibodies including anti-Ro/SSA, anti-La/SSB, anti-nuclear antibodies. In vitro studies demonstrated that LAMP3 expression induces epithelial cell dysfunction leading to apoptosis. Interestingly, LAMP3 expression resulted in the accumulation and release of intracellular TRIM21 (one component of SSA), La (SSB), and α-fodrin protein, common autoantigens in Sjögren’s syndrome, via extracellular vesicles in an apoptosis-independent mechanism. This study defines a clear role for LAMP3 in the initiation of apoptosis and an independent pathway for the extracellular release of known autoantigens leading to the formation of autoantibodies associated with this disease.

ClinicalTrials.gov Identifier: NCT00001196, NCT00001390, NCT02327884.

## Introduction

Primary Sjögren’s syndrome (pSS) is one of the most common autoimmune diseases, and has a strong female bias (female:male; 9:1)^1^. pSS is characterized by dry mouth, dry eyes, lymphocytic infiltration of the affected glands (e.g., sialadenitis and dacryoadenitis), and the presence of serum autoantibodies, such as anti-Ro/SSA, anti-La/SSB, rheumatoid factor and anti-nuclear autoantibodies (ANA)^2,3^. Of these, antibodies to Ro/SSA and La/SSB are biomarkers of this syndrome and are found in approximately 70% and 50% of pSS patients, respectively^2–4^. Studies of biobanked serum samples suggest that 66–81% of Sjögren’s patients have autoantibodies many years prior to the onset of disease^5,6^.

In 2005, Ramos-Casals and Font proposed a model of primary SS based on the existence of an altered immune system that cannot discriminate between self and non-self antigens^7^. This model has evolved to suggest the disease is initiated by a combination of genetic and environmental factors. One potential initiating event of the disease may be an abnormal immune response against a cellular or viral antigens resulting in the production of pro-inflammatory cytokines, infiltration of immune cells and formation of characteristic inflammatory foci, and decreased gland function. Alternatively, mechanisms involving tissue damage (such as apoptosis) could lead to autoantibody formation and loss of exocrine gland function.

Apoptosis has recently been identified as a mechanism of cell death in salivary gland epithelial cells from pSS patients and in mouse models of pSS, in which DNA fragmentation detected by TUNEL assays, increased GADD153 expression, and decreased Del-1 expression^8,9^ were observed. However, our understanding of the potential mechanisms associated with the increase in apoptosis and the development of autoimmunity, and how this relates to the diverse set of symptoms associated with pSS, is limited.

Lysosomal proteins are key components involved in antigen presentation and cell survival^10,11^. Unlike canonical lysosome-associated membrane proteins such as LAMP1 and LAMP2, LAMP3 is tightly regulated and its expression is induced during infection^12^. LAMP3 expression is cell-specific and found in dendritic cells and type II pneumocytes^13,14^. Recent studies also suggest that LAMP3 expression is limited to the membrane of MHC class II molecule intracellular storage compartment (MIIC) and tubules/vesicles^13,15^. Mice immunized with LAMP3/HIV-1 p55Gag Ag chimera DNA plasmid show a stronger Th type 1 response compared with HIV-1 p55Gag Ag DNA plasmid immunized mice suggesting an important role for LAMP3 in antigen presentation^16^. LAMP3 also plays a role in autophagy in which LAMP3 knockdown in cells inhibits the autophagic progression leading to death by apoptosis^17,18^. Lysososmal associated cell death is likely driven by lysosomal membrane permeabilization (LMP) that is triggered by the release of cathepsins into the cytoplasm has been previously reported^19^.

Unbiased transcriptome analysis can be a powerful approach for understanding the pathophysiology of diseases. Although single transcriptomic studies show promise for identifying disease markers, aggregation of multiple transcriptome data sets provides more statistical power to detect important disease-specific alterations and reduces the likelihood of spurious results for complex diseases such as cancer and preeclampsia^20,21^. In this study aggregated data from multiple transcriptomic studies were used to analyze minor salivary gland (MSG) biopsies from patients with Sjögren’s syndrome (SS) compared with control groups. This analysis combined with immunofluorescences identified increased expression of LAMP3 in both infiltrating lymphocytes and salivary gland ductal and acinar epithelia. After stratification of pSS patients based on their clinical symptoms, LAMP3 expression was associated with the presence of serum autoantibodies. In vitro studies identified that LAMP3 expression induced activation of caspase-3 activity and led to apoptosis of transfected cells. In addition this increase in LAMP3 led to the release of common Sjögren’s syndrome autoantigens via extracellular vesicles in caspase-independent manner, identifying a potential mechanism involved in their antigenicity.

## Results

### LAMP3 expression is increased in minor salivary glands of SS patients and associated with the presence of serum autoantibodies

Several independent transcriptomic studies investigating gene expression changes in the salivary glands of SS patients have been reported^22–25^. Aggregated and integrated analysis of the available public data was employed to identify common transcriptomic changes in the disease and reduce experimental bias. Here microarray data from three studies investigating MSG biopsies, which met our inclusion criteria were aggregated and used to compare SS patients with three healthy or disease controls groups consisting of healthy volunteers, non-SS patients with sicca symptoms, and patients with IgG4-related disease. This composite analysis identified 279 common differentially expressed genes (Supplementary Table S1)^22–24^. Ingenuity pathway analysis demonstrated enrichment of pathways associated with interferon signaling, pattern recognition of pathogens, RhoGDI signaling, and apoptosis, some of which have been previously reported associated with SS (Supplementary Table S2). Decreased calcium signaling, G protein-coupled receptor mediated nutrient sensing, and amyloid processing were also found in the SS patients compared with controls (Supplementary Table S2).

**Figure 1:**
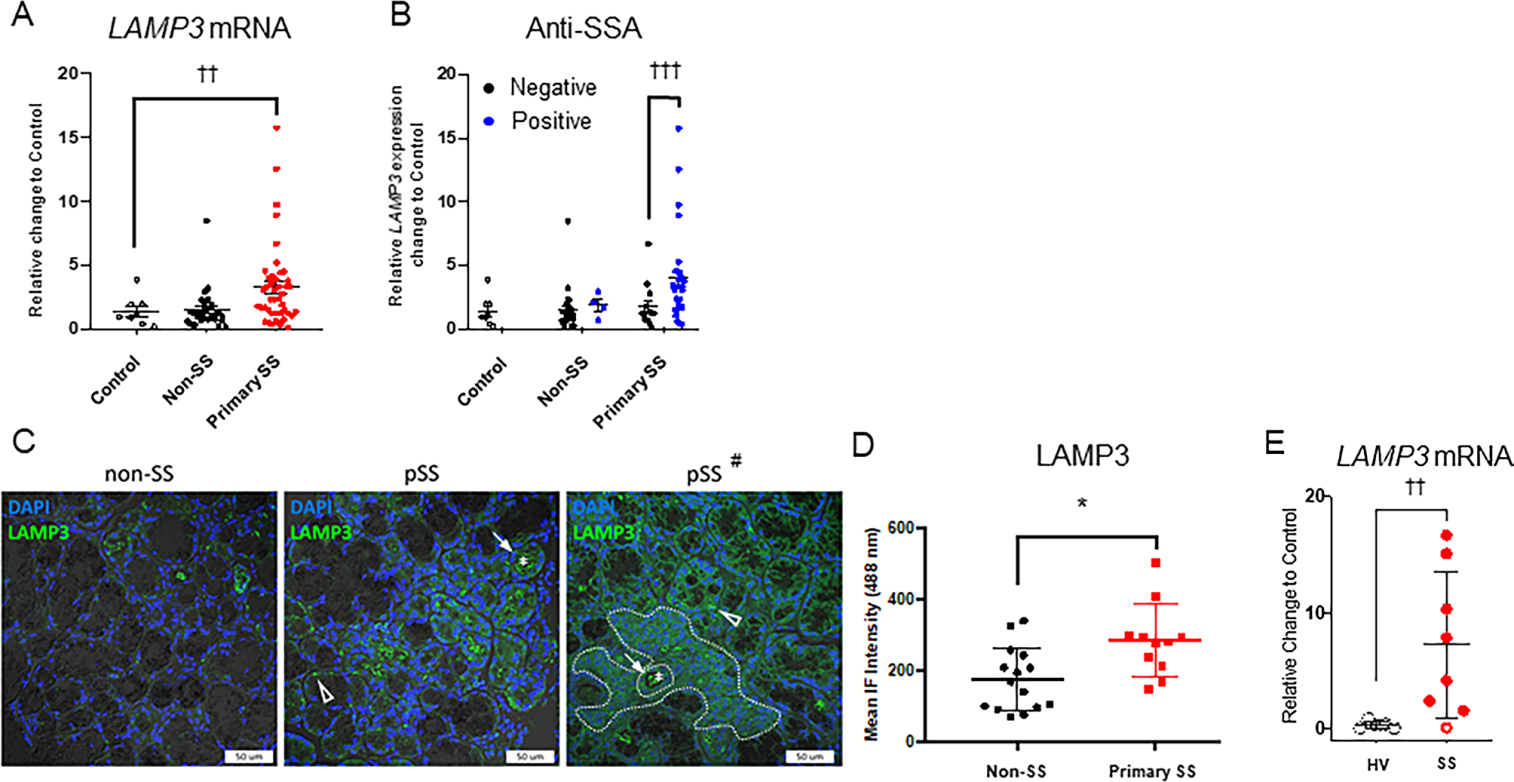
LAMP3 expression is increased in Sjögren’s syndrome patients, and increased expression is associated with serum autoantibodies. (**A**) LAMP3 mRNA expression levels in controls (healthy volunteers; open circle), non-SS (black dot) and primary SS (red dot) patients (Mean ± SD). (**B**) LAMP3 mRNA expression levels in controls, non-SS patients and SS patients, with or without presence of serum anti-SSA antibody (Mean ± SD). (**C**) Representative confocal immunofluorescent (IF) images (×40 magnification) of LAMP3 protein expression in (left image) a patient who does not meet SS criteria (non-SS), (middle image) a patient who meets criteria for primary SS (pSS) with median LAMP3 expression, (right image) and a patient (pSS#) with a characteristic lymphocytic focus adjacent to normal salivary gland epithelial tissue (ducts and acini). LAMP3 positive aggregates are present in acinar (open triangle) and ductal epithelia (arrow), the luminal debri (asterisks), and the lymphocytic foci (dashed outline). (**D**) IF intensity for LAMP3 protein expression in MSG biopsies from non-SS and pSS patients (Mean ± SD). (**E**) Differential expression of *LAMP3* in human primary salivary gland epithelial cells (pSGECs) established from 7 SS patients and 8 healthy volunteers was analyzed by using RT-qPCR (open circle: SSA and SSB negative, closed circle: SSA and/or SSB positive). *P < 0.05, ANoVA; ^††^*P* < 0.01, ^†††^*P* < 0.001, unpaired Student’s *t*-test.

One of the most significantly upregulated genes, excluding interferon associated genes, was *LAMP3.* Confirmatory qRT-PCR experiments demonstrated increased *LAMP3* mRNA expression in a separate cohort of MSG samples from SS patients compared with healthy volunteers (HV) or MSG samples from non-SS patients with other autoimmune diseases (Fig. 1A).

Analysis of *LAMP3* mRNA expression with clinical covariates demonstrated that high *LAMP3* expression (defined as > 2SD over the mean level in healthy volunteers) is associated with the presence of serum autoantibodies. Specifically, a significant positive association with increased *LAMP3* mRNA expression was found with anti-SSA autoantibody seropositivity in pSS (Fig. 1B) as well as anti-SSB, ANA, total IgG, and focus score (Supplementary Figure S1A–D). No association was found with other immune components such as complement C4 or C3, beta2 microglobulin levels.

Based on the increased *LAMP3* mRNA, we immunolocalized LAMP3 positive cells within the salivary glands. Confocal immunofluorescent imaging demonstrated elevated expression of LAMP3 protein in both the epithelial and infiltrating lymphocytic cell compartments of SS MSG biopsies compared with non-SS control MSG biopsies (*P* < 0.05) (Fig. 1C,D). Since LAMP3 protein expression was not restricted to infiltrating or resident immune cells, the differential epithelial expression of *LAMP3* using primary salivary gland epithelial cells (pSGECs) derived from healthy volunteers and SS patients was confirmed. Interestingly, *LAMP3* mRNA was significantly increased in pSGECs derived from SS subjects compared with HV (*p* < 0.01) (Fig. 1E). These findings suggest an important link between increased LAMP3 expression and classical autoantibody seropositivity in SS.

### LAMP3 overexpression induces expression and redistribution of SS autoantigens

LAMP3 expression has previously been reported in dendritic cells but little is known about its role in epithelia. To examine the effect of LAMP3 on epithelial cell function, we established LAMP3-overexpressing HSG (HSG LAMP3-OE) and A253 (A253 LAMP3-OE) cells by transfection with the LAMP3 encoding plasmid, pME18S-LAMP3. Confocal immunofluorescent microscopy was also used to investigate the effect of LAMP3 overexpression on TRIM21/Ro52 and SSB expression and localization. Ro60 was not investigated since 90% of anti-Ro52 positive patients are Ro60 positive^26^.

**Figure 2:**
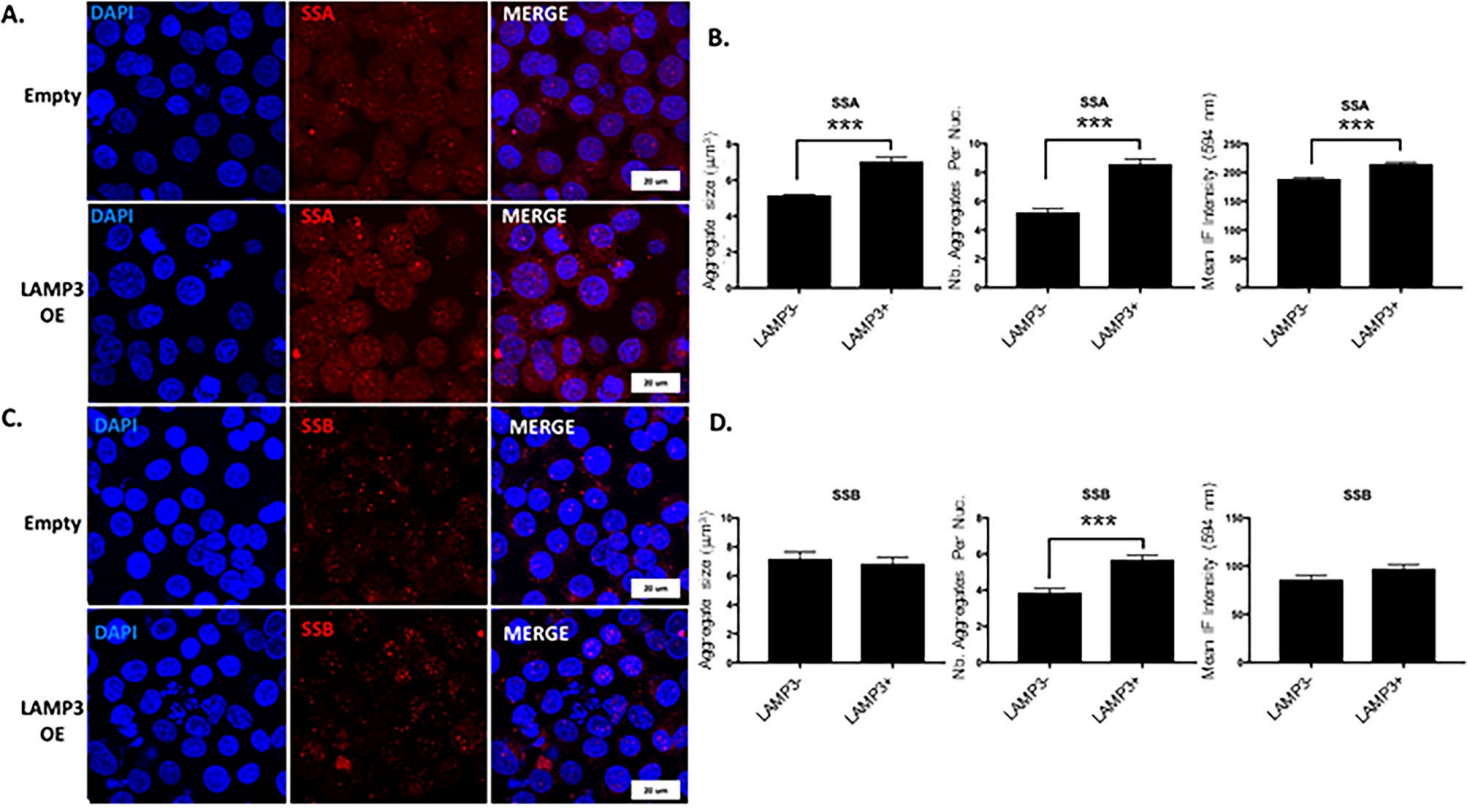
LAMP3 overexpression increases TRIM21 expression and number and size of nuclear aggregates. Representative confocal immunofluorescent (IF) images (60X objective) of (**A**) TRIM21/SSA and (**C**) SSB protein expression in control HSG cells (empty) and LAMP3-overexpressing (OE) HSG cells. Nuclear aggregate size, number of nuclear aggregates per nuclei, and total IF intensity for (**B**) TRIM21/SSA and (**D**) SSB in control and LAMP3 OE cells. TRIM21/SSA aggregate size: N = 144 cells (control) and 558 cells (LAMP3-OE); TRIM21/SSA aggregate nember per nuclei: N = 149 cells (control) and 84 cells (LAMP3-OE); TRIM21/SSA total IF intensity: N = 177 cells (control) and 146 cells (LAMP3-OE). SSB aggregate size: N = 359 cells (control) and 430 cells (LAMP3-OE); SSB aggregate nember per nuclei: N = 130 cells (control) and 117 cells (LAMP3-OE); SSB total IF intensity:N = 146 cells (control) and 154 cells (LAMP3-OE). ***P < 0.001, ANoVA. Values shown are mean ± SD.

TRIM21 immunofluorescence demonstrated a diffuse, fine cytoplasmic and nuclear staining with scattered cells demonstrating a punctate nucleolar-like pattern in control HSG cells. In contrast, LAMP3 transfected cells showed nuclear redistribution of TRIM21 (multiple coarse dots spanning throughout the nucleus) and increased overall expression of TRIM21, including significant increases in the size of aggregates, the number of nuclear TRIM21 aggregates, and overall expression (*P* < 0.001) (Fig. 2A,B). SSB demonstrated a similar staining pattern as TRIM21 in control cells and similar nuclear redistribution in LAMP3 overexpressing cell; although overall expression was less compared with TRIM21. LAMP3 overexpression increased the number of nuclear aggregates of SSB (*P* < 0.001), but did not affect the size; and there was a trend towards increased expression (Fig. 2C,D). In addition, LAMP3-OE cells commonly showed changes in nuclear structure associated with late apoptosis and apoptotic nuclear debris were commonly visible. Furthermore, the pattern of nuclear redistribution of TRIM21 (and to a lesser degree SSB) shown by immunofluorescence is indicative of LAMP3-specific initiation of apoptosis and are similar to apoptitic changes previously reported in SS patients^27^.

### LAMP3 induces apoptosis via caspase pathway

**Figure 3:**
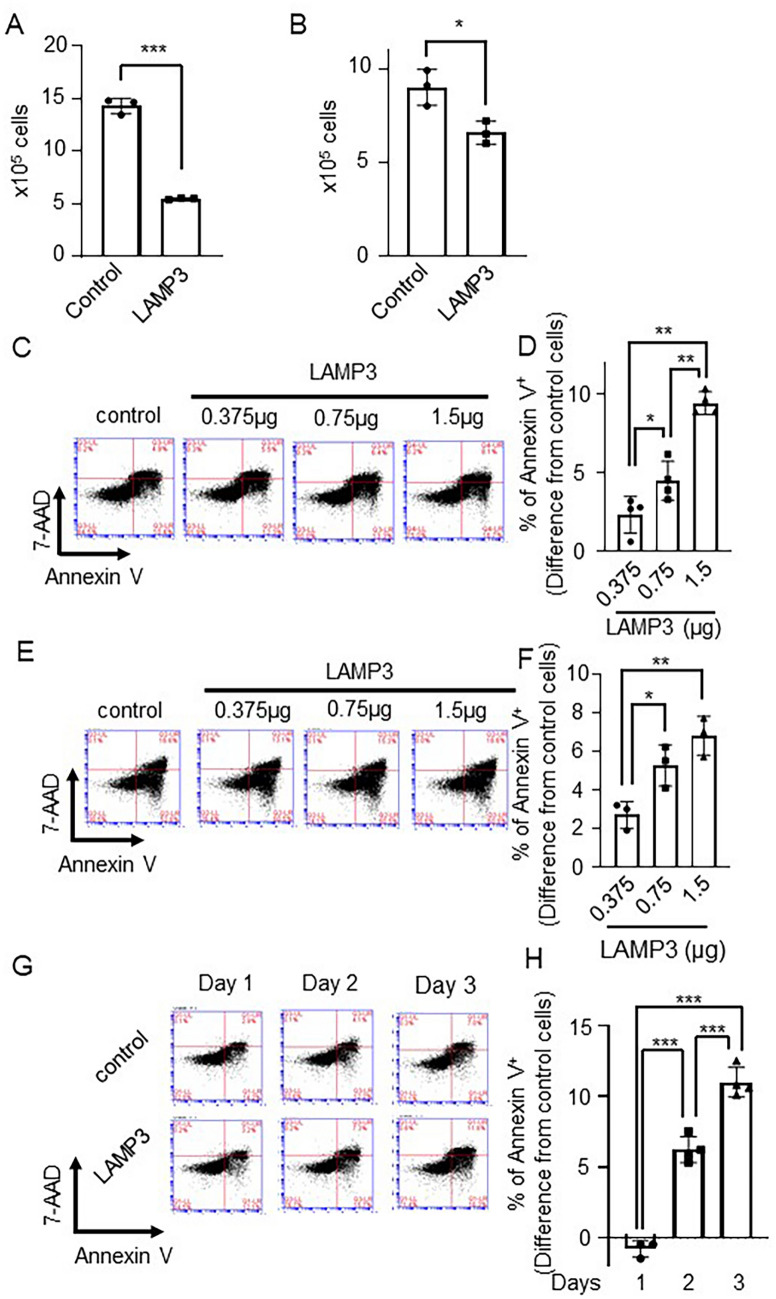

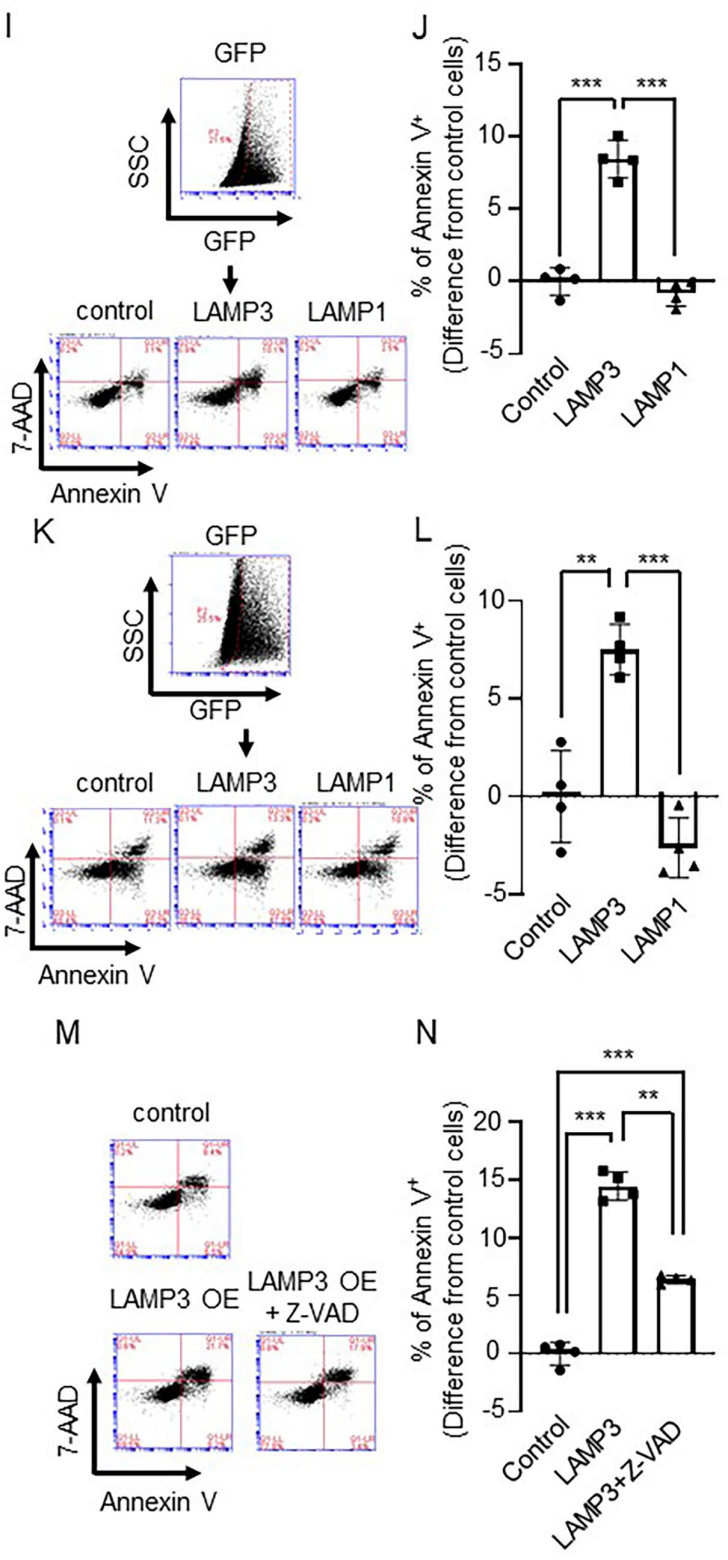
LAMP3 inhibits cell growth and induces caspase-dependent apoptosis. (**A**) HSG and (**B**) A253 cells were transfected with 1.5 μg pME18S-empty or pME18S-LAMP3 plasmid, and then 2 × 10^5^ cells of each cells were re-plated 24 h post-transfection. The number of cells was counted 96 h after re-plating with Countess Automated Cell Counter (N = 3 independent experiment and 2 technical replicates of each experiment). (**C**,**D**) HSG cells and (**E**,**F**) A253 cells were transfected with different concentrations of pME18S-LAMP3 plasmid or a pME18S-empty plasmid as control. Seventy-two hours post-transfection, apoptotic cells were counted by using flow cytometry with APC Annexin V/7-AAD. Difference from control cells is shown (N = 4). (**G**,**H**) Number of apoptotic cells in control and LAMP3-overexpressing (OE) HSG cell cultures 24, 48 and 72 h after transfection, assessed by flow cytometry using APC Annexin V/7-AAD. Difference from control cells is shown (N = 3). (**I**,**J**) Number of apoptotic cells in GFP^+^ control, LAMP3-OE and LAMP1-OE HSG cell cultures 48 h after transfection, assessed by flow cytometry using APC Annexin V/7-AAD. Difference from control cells is shown (N = 4). (**K**,**L**) Number of apoptotic cells in GFP^+^ control, LAMP3-OE and LAMP1-OE A253 cell cultures 48 h after transfection, assessed by flow cytometry using APC Annexin V/7-AAD. Difference from control cells is shown (N = 4). (**M**,**N**) Control and LAMP3-OE HSG cells were incubated with or without 20 μM Z-VAD-FMK (Z-VAD) for 20 h. Extent of apoptosis was determined by flow cytometry using APC Annexin V/7-AAD. Difference in rate of Annexin V^+^ cells in LAMP3-OE cell culture treated with or without 20 μM Z-VAD from that in control cell culture is shown (N = 4). *P < 0.05, **P < 0.01, ***P < 0.001, unpaired Student’s *t*-test. Values shown are mean ± SD.

Analysis of the growth characteristics of LAMP3 overexpressing cells showed a significantly reduced cell growth in both HSG and A253 cell lines compared with control cells transfected with an empty vector, when determined by trypan blue exclusion assay (HSG: 60% decrease, *P* < 0.001; A253: 30% decrease, *P* < 0.05) (Fig. 3A,B).

To determine whether the changes in cell growth rates were the result of alterations in cell cycle or increased apoptosis, the cell cycle in control and HSG and A253 LAMP3-OE cells was analyzed by Propidium Iodide (PI) and flow cytometry. LAMP3 expression in HSG or A253 cells induced a small but statistically significant change in the percentage of cells in different phases of the cell cycle compared with control treated cells (Supplementary Figure S2). However, these changes were likely too small to account for the observed change in growth.

To further understand the effect of LAMP3 increased expression in epithelial cells, markers of apoptosis were studied. HSG and A253 LAMP3-OE cells exhibit a significant time- and dose-dependent increase in the number of Annexin V^+^ cells compared with control transfected cells as measured by Annexin V/7-AAD staining and flow cytometry (Fig. 3C–H). Similar result was found in NS-SV-AC cells (Supplementary Figure S3). To test whether these results were specific to LAMP3 overexpression, HSG and A253 cells were co-transfected with a GFP reporter plasmid and either a LAMP3 or LAMP1 expression vectors and the number of apoptotic cells in the GFP^+^ population were counted. The number of apoptotic cells were significantly increased in the LAMP3 co-transfected GFP^+^ cells compared with GFP^+^ control cells and LAMP1 co-transfected GFP^+^ cells at both early and late stages of apoptosis (Fig. 3I–L). Furthermore, no differences in the number of apoptotic cells were found between the LAMP1-OE cells and control cells for both cell lines (Fig. 3I–L) suggesting the increase in apoptosis was specific to LAMP3 expression.

Caspases are well-known initiators of apoptosis^28,29^. Treatment of LAMP3-OE cells with the pan-caspase inhibitor, Z-VAD, which also inhibits caspase-3^30^, showed a significant decrease in apoptosis compared with LAMP3-OE controls (LAMP3 + Z-VAD vs. LAMP3: 6.4 ± 0.3% vs. 14.5 ± 1.2%, *P* < 0.01) (Fig. 3M,N). Taken together, these results imply that LAMP3-associated decrease in cell growth is caused by caspase-dependent induction of apoptosis.

### LAMP3 induces apoptosis via caspase-3 independently of endoplasmic reticulum stress

**Figure 4:**
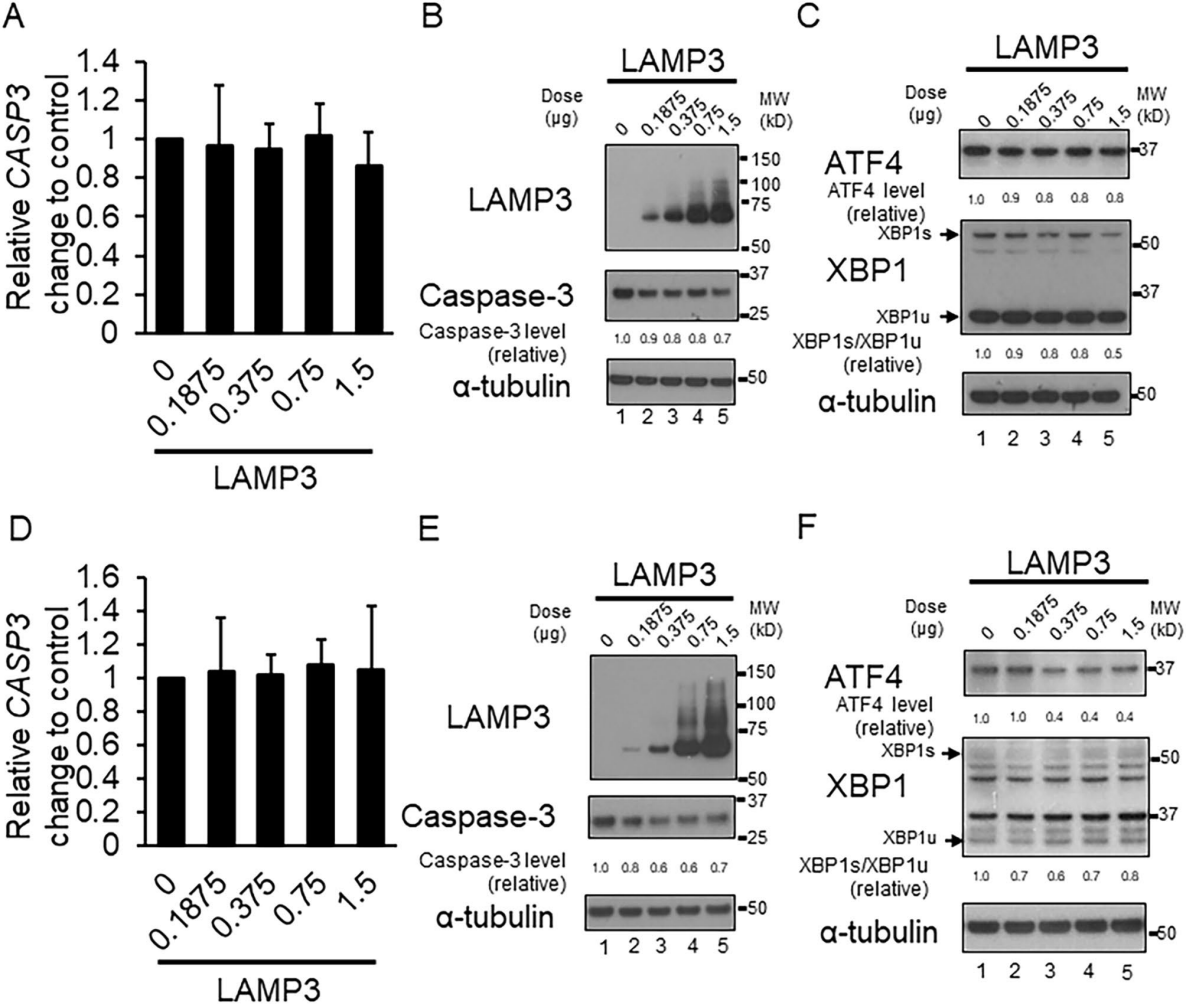
LAMP3-induced activation of caspase-3 is independent of endoplasmic reticulum stress. Caspase-3 (CASP3) mRNA expression levels were measured by qRT-PCR in control and LAMP3-overexpressing (OE) (**A**) HSG and (**D**) A253 cells. Western blot analysis was performed to measure (**B**,**E**) caspase-3 protein, (**C**,**F**) ATF4, spliced XBP1 (XBP1s) and unspliced XBP1 (XBP1u) protein levels in HSG and A253 control and LAMP3-OE cells, respectively. All ratios are relative value compared with control. Uncropped images are provided in Supplementary Figure S4.

LAMP3-OE cells were also used to investigate the effect of LAMP3 on caspase-3 expression, caspase-3 mRNA and protein levels. Athough no difference in caspase-3 mRNA expression between LAMP3-OE and control cells (Fig. 4A,D) was found, caspase-3 protein expression was decreased in LAMP3-OE cells compared with control cells (Fig. 4B,E), suggesting that LAMP3 overexpression alters caspase-3 cleavage and activation. Endoplasmic reticulum (ER) stress is often linked to apoptosis via caspase activation^31,32^. Therefore, LAMP3-induced ER stress was monitored by following the change in expression of ATF4 and activation of XBP-1, well-known markers of ER stress. No increase in expression of ATF4 or activation of XBP1 was observed in LAMP3-OE cells compared with control cells (Fig. 4C,F), suggesting that LAMP3 induced apoptosis was independent of ER stress.

### LAMP3 induces accumulation and release of autoantigens via extracellular vesicles

Extracellular vesicles (EVs), including apoptotic bodies, are thought to be sources of neoantigens within the pathogenesis of autoimmune diseases^33–36^. Based on the finding that LAMP3 expression in the MSG biopsies from SS patients impacted the survival of the cells by inducing caspase-mediated apoptosis and changed the expression and localization of key autoantigens associated with SS (Fig. 2). We hypothesized that LAMP3-induced release of TRIM21/SSA and La/SSB might be a source of autoantigens and account for the association between MSG LAMP3 expression and the presence of autoantibodies in pSS patients.

**Figure 5:**
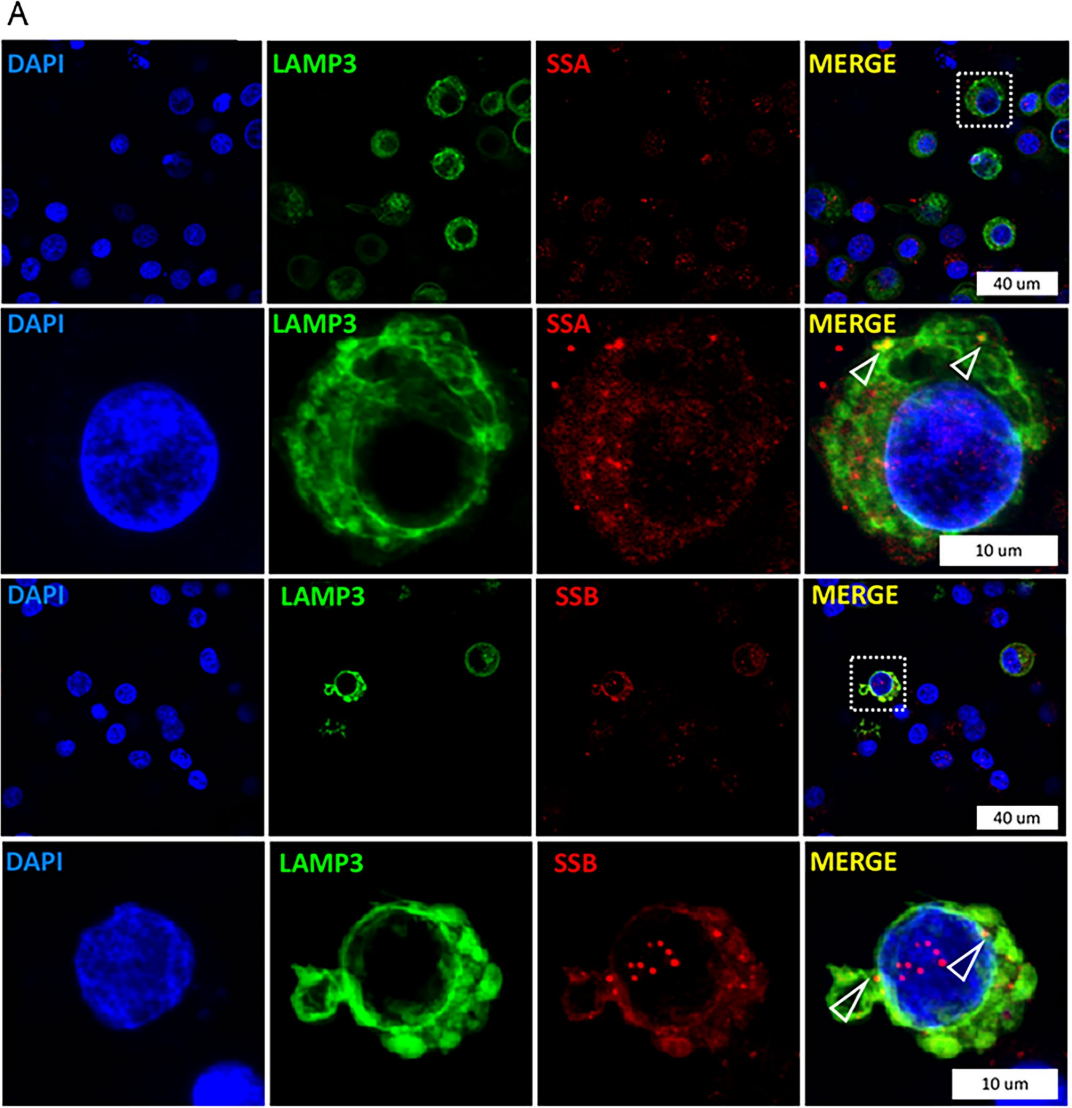

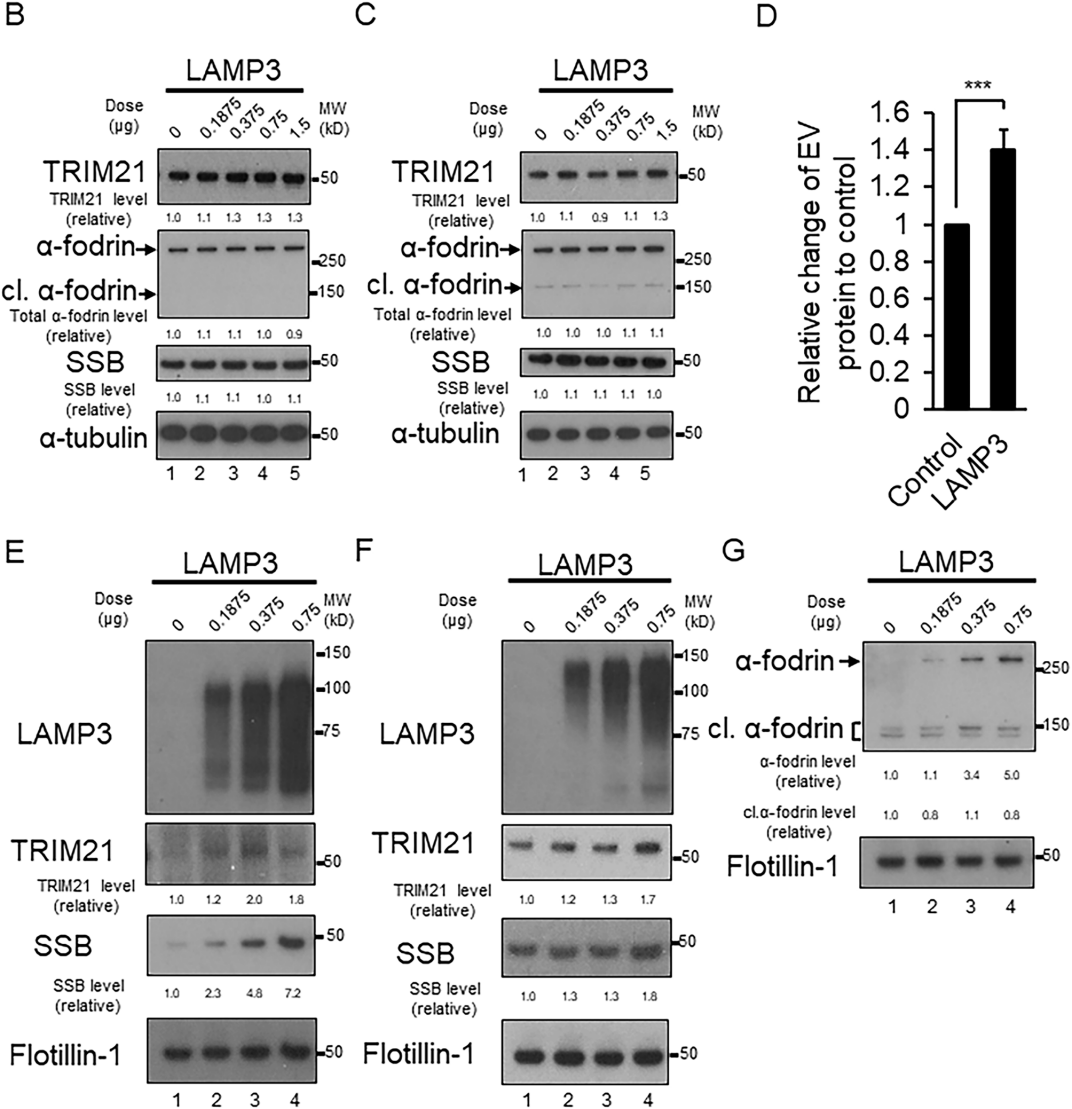
LAMP3-induced accumulation of autoantigens and release via extracellular vesicles. (**A**) Images were collected by immunofluorescent microscopy at 100X magnification. LAMP3-overexpressing (OE) cells show a membranous and vesicular pattern of LAMP3 expression. Some of the vesicles appear just below or budding from the plasma membrane and colocalize with TRIM21/SSA and SSB (open triangles). TRIM21/SSA, SSB, α-fodrin and cleaved (cl.) α-fodrin protein levels in control and LAMP3-OE cells. Rows 2 and 4 are further enlarged images of specific cells shown in row 1 and 3. The specific cells in the merged image are boxed for clarity. Indicated protein expression in (**B**) HSG and (**C**) A253 cells, as determined by Western blotting. (**D**) Protein concentration in mixture of extracellular vesicles (EVs) isolated from control and LAMP3-OE HSG cells. Data are presented as relative change in expression compared with control. Western blotting analysis of TRIM21/SSA and SSB in EVs isolated from control and LAMP3-OE (**E**) HSG and (**F**) A253 cells. (**G**) Western blotting of α-fodrin and cl. α-fodrin in EVs from control and LAMP3-OE HSG cells. Protein levels were normalized to α-tubulin or flotillin-1 level. Uncropped images are provided in Supplementary Figure S5. ***p < 0.001, unpaired Student’s *t-*test.

Using confocal immunofluorescent microscopy, a membranous expression pattern of LAMP3, particularly in structures appearing to bud from the plasma membrane, was observed with the appearance of EVs. In LAMP3-OE cells a clear colocalization of LAMP3 with TRIM21/SSA and SSB was observed within EVs (open triangles, Fig. 5A). Western blotting showed a difference in TRIM21 expression compared with control cells. However, no difference in the expression of SSB or α-fodrin was observed (Fig. 5B,C). Analysis of the protein content of the EVs isolated from HSG LAMP3-OE showed an increase compared with EVs isolated from control cells (Fig. 5D). Moreover, western blotting of the EVs showed a significant increase in the levels of TRIM21, SSB, and α-fodrin in EVs from LAMP3-OE cells compared with control cells (Fig. 5E–G). However, no difference was detected in the amount of cleaved α-fodrin protein in the EVs from LAMP3-OE cells compared with control cells (Fig. 5G). These results suggest that in addition to the increase in LAMP3-associated apoptosis, LAMP3 over expression induces the release of autoantigens from the cell.

### LAMP3-induced autoantigens release is independent of apoptosis

**Figure 6:**
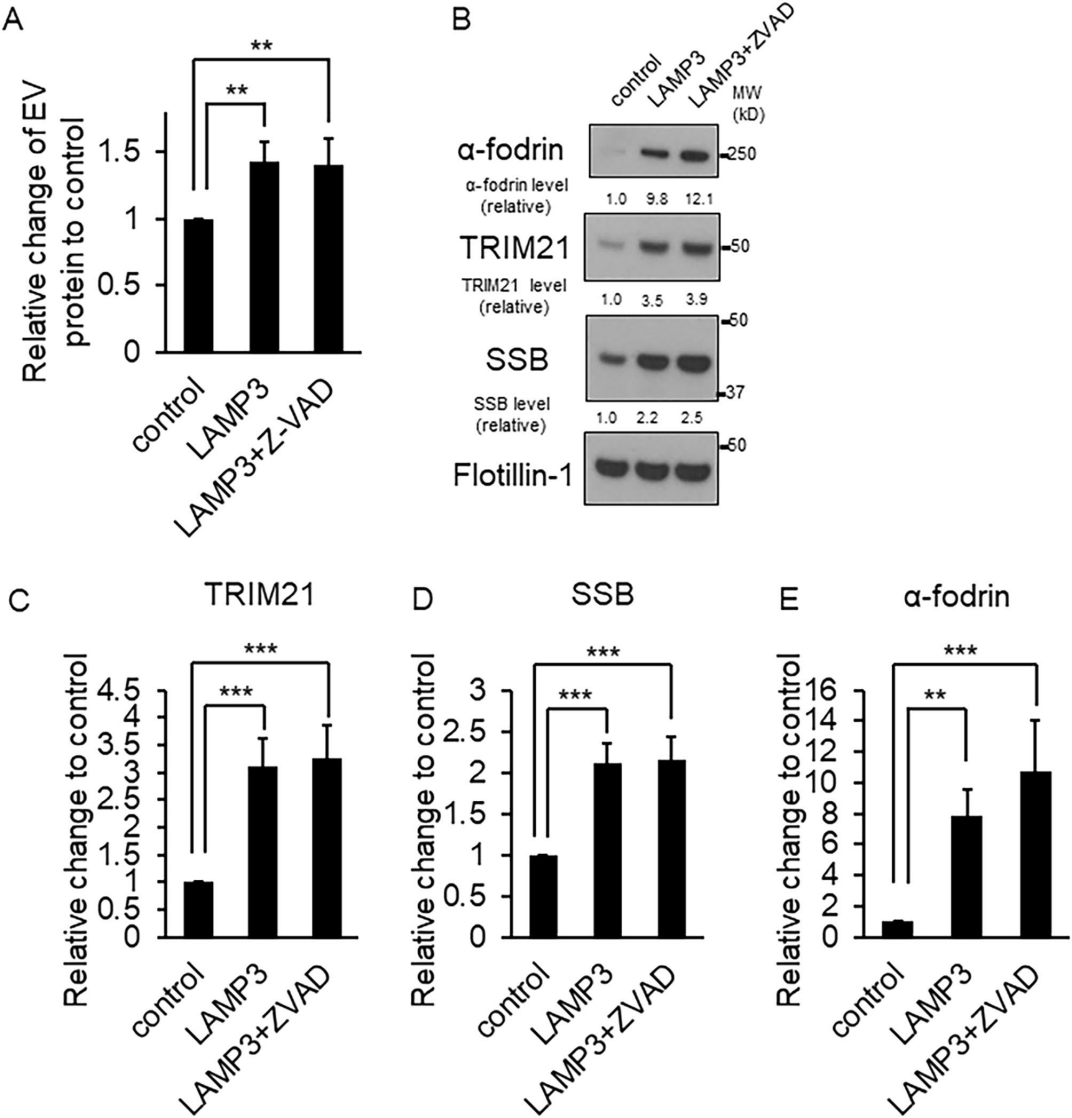
LAMP3-induced autoantigens release was independent of apoptosis. (**A**) Protein concentration in mixture of extracellular vesicles (EVs) isolated from control and LAMP3-OE HSG cells treated with or without Z-VAD. Data are presented as relative change in expression compared with control cells. (**B**) Western blotting analysis of TRIM21/SSA, SSB and α-fodrin in EVs isolated from HSG control and LAMP3-OE cells treated with or withour Z-VAD. Protein levels of (**C**) TRIM21/SSA, (**D**) SSB and (**E**) α-fodrin were normalized to flotillin-1 level, and the value was plotted. Uncropped images are provided in Supplementary Figure S6. **p < 0.01, ***p < 0.001, unpaired Student’s *t-*test.

One important question was whether LAMP3-associated apoptosis was responsible for antigen release. To clarify this, HSG LAMP3-OE cells were treated with Z-VAD, an inhibitor of apoptosis, and the release of autoantigens in EVs was measured. No siginificantly difference was found in the protein content of the EVs isolated from HSG LAMP3-OE cells treated with Z-VAD compared with EVs from LAMP3-OE control cells, in which both treated and untreated HSG-OE cells showed increased EVs compared with control cells (Fig. 6A). Western blotting also showed no difference in autoantigens, TRIM21, SSB and α-fodrin protein levels in the EVs isolated from HSG LAMP3-OE cells treated with Z-VAD compared with HSG LAMP3-OE control cells (Fig. 6B–E). These results suggested that LAMP3-induced autoantigen release is independent of apoptosis.

## Discussion

Sjögren’s syndrome is a complex disorder of uncertain etiology and pathogenesis. Patients exhibit a heterogenous clinical presentation with multiple molecular subsets of patients which is likely responsible for the lack of universal predictive biomarkers or effective treatments. However, one common feature shared in approximately 70% of the patients is the presence of specific autoantibodies. To gain further insight into the drivers of disease, we employed aggregated transcriptome analysis using previously published transcriptomic studies to analyze the MSG biopsies from SS patients and controls to illuminate common genes and pathways. From this approach, we found increased LAMP3 mRNA expression in a subset of pSS patients. Unexpectedly, we found that patients exhibiting increased LAMP3 expression also were patients with anti-SSA and anti-SSB autoantibodies. Futhermore, this increase could be observed in primary cultures from the patients suggesting SS-related transcriptional reprogramming may not entirely dependent upon immune microenvironment stimulation. Our investigation of the biological impact of LAMP3 upregulation in epithelial cells suggests that it can initiate cell death and apoptosis independent autoantigen release.

Serum autoantibodies against several intracellular proteins (e.g., TRIM21 (Ro52), La/SSB) are found in approximately 70% of pSS patients who meet diagnostic criteria^3,4^. Given the commonality of these serum autoantibodies across a majority of patients, it is likely that these proteins play key roles in the pathogenesis of SS. It has been proposed that phagocytosis of apoptotic bodies and debris leads to the presentation of autoantigens and subsequently to the induction of autoimmunity. However, the mechanism responsible cell death and the resultant release of autoantigens that triggers these aspects of the disease remains largely unknown.

Although anti-Ro/SSA and anti-La/SSB autoantibodies are sometimes found in other autoimmune diseases such as systemic lupus erythematosus, a correlation with a specific differentially expressed gene has not been established. This may be the result of the localized nature of SS tissue destruction to secretory epithelial tissues and limited access to the affected tissue. Our key finding represents a previously unrecognized connection between autoantibody formation, lysosomal protein expression, and apoptosis. Further inquiry into changes in the expression of lysosomal proteins and the induction of autoantibodies in other autoimmune diseases is warranted.

In this study we identified a novel mechanism for apoptosis induction in the course of developing autoimmunity. In turn apoptosis can induce the cell surface expression of autoantigens such as TRIM21, La/SSB, and DNA, and the release of autoantigens. TRIM21 is an attractive protein for the development of autoimmunity given its role in the cellular innate immune response and surveillance to intracellular pathogens such as viruses. TRIM21 recognizes intracellular pathogens, binds to the Fc portion of the human IgG-bound to pathogens marking them for degradation and initiates the innate immune response^37,38^. Additional studies indicate that immunizing with antigens such as TRIM21, La/SSB, and α-fodrin leads to autoantibody production and the autoimmune disease^39–42^. Additionally, cell surface autoantigen expression induced by apoptosis elevates autoantibody production via antigen presenting cells (APCs)^43^. Moreover, apoptotic bodies can stimulate dendritic cells, and immunizing with apoptotic cells leads to auto-IgG production including autoantibodies against TRIM21 and SSB^44^. LAMP3 expression increased the accumulation and aggregation of TRIM21, and the release of TRIM21, SSB, and α-fodrin (Figs. 2, 5A–G), suggesting that LAMP3 can function as a potent initiator of the release of neoantigens via EVs. Taken together, these results support the idea that LAMP3 plays an important role in the activation of autoimmune responses through accumulation of TRIM21, and the release of TRIM21, La/SSB, and α-fodrin as autoantigen via EVs independent of apoptosis.

Endoplasmic reticulum stress has been implicated in the pathogenesis of a wide variety of human diseases, including inflammatory disease^45^ and autoimmune disease^46^. Increased ER stress markers in MSG biopsies from SS patients have been reported^47^. The unfolded protein response (UPR) is activated by, and actively modulates, ER stress, to maintain homeostasis and energy balance within cells. LAMP3 is activated downstream of the PERK-ATF4 signaling pathway, which is part of the UPR pathway, and is induced by ER stress^48^. Although ATF4 expression was not increased by LAMP3 expression in this study, we postulate that it could be increased by the same environmental stimuli that are likely responsible for the resultant increase in LAMP3 expression and represents a central aspect to the initiation of SS. This study showed a clear link between caspase-dependent apoptosis and LAMP3 expression. Further studies are needed to investigate the effect of LAMP3 on the balance between apoptotic and cell survival pathways.

LAMP3 overexpressing cells showed cell death typical for apoptosis. LAMP3 is classically associated with the lysosome, a main organelle central to autophagy. This cell survival mechanism is also known to kill cells under certain conditions in a process called autophagic cell death, which involves pathways and mediators different from those of apoptosis^49–51^. Although our data supports an apoptotic pathway in SS tissue destruction, it is possible that LAMP3 expression may also affect autophagy, leading to apoptotic cell death. Cross talk between apoptosis and autophagy via the lysosome maybe central to the underlying pathogenesis in Sjögren’s syndrome.

This study shows that LAMP3 expression contributes to the induction of apoptosis, which has long been associated with SS by an unknown mechanism. Interestingly the LAMP3 associated release of autoantigens is independent of apoptosis. The results presented here suggest a previously unrecognized context for autoantigen presentation and defines a central role for LAMP3 in pSS. Autoantibody formation is not unique to Sjögren’s syndrome and this mechanism of autoantigen release maybe at work in other autoimmune diseases. Currently therapeutics are being developed to block the caspase-dependent apoptosis. Additional therapeutic targets may emerge by investigating how LAMP3 is specifically inducing apoptosis and preventing the development of autoantibodies following antigen release.

## Materials and methods

### Study selection and data aggregation

Previously published microarray studies on Sjögren’s syndrome were reviewed using inclusion and exclusion criteria^22–24^. Inclusion criteria were for three or more patients, array platforms with more than 40,000 genes, available raw data sets, clearly defined diagnostic criteria based on AECG 2002 criteria^4^, and use of high quality RNA isolated from minor salivary glands. Exclusion criteria included low density arrays, samples on mice and those that used blood samples.

Selected data sets were collected from NCBI gene expression omnibus (GEO) using GeneSpring Multi-Omic Analysis version 14.9^52^. A total of three data sets representing four clinically-defined subsets of patients were identified in GEO: GSE40568, GSE23117, GSE127952.

Each data set was treated as a separate differential gene expression analysis experiment and was analyzed without further normalization. Differentially expressed genes were then aggregated by GeneSpring and used as input for Ingenuity Pathways Analysis (IPA) to identify common genes and pathways.

### Clinical studies

MSG biopsies used in the transcriptional studies were obtained from two centers: the Sjögren’s Syndrome Clinic at the National Institute of Dental and Craniofacial Research (NIDCR), National Institutes of Health (NIH), in Bethesda, MD, and from Clinics Hospital of the Medical School of Ribeirao Preto (CHRMSRP), University of São Paulo, São Paulo, Brazil. Primary salivary gland epithelial cells (pSGECs) were drived from MSG biopsies obtained from human subjects in the Sjögren’s Syndrome Clinic at NIDCR. All studies using human tissues were carried out in accordance with approved NIH guidelines. Subjects under study provided informed consent prior to the initiation of any study procedures. NIH human tissues were obtained from NIH Institutional Review Board (IRB) approved protocols (ClinicalTrials.gov Identifiers: NCT00001390, NCT02327884, or NCT00001196) in the Sjögren’s Syndrome Clinic at the NIDCR, NIH in Bethesda, MD. Likewise, CHMSRP human tissues were obtained from subjects who provided informed consent to an IRB-approved protocol approved by the Brazilian Committee of Ethics in Research CAAE: 37688914.2.0000.5440). American-European Consensus Group (AECG) criteria was used to classify subjects as SS^4^. Control patients in this study responded negatively to a questioning regarding the presence of oral symptoms of xerostomia or xeropthalmia, as per the European American Criteria for the diagnosis of Sjögren’s syndrome. A table of patient information used for stratification can be found in Table 1. A table of patient information used in the microarray study is provided in Supplementary Table S3. See “Supplementary Methods” for RNA isolation.

**Table 1:** Clinical feature of non-SS and SS samples.

Subjects	Age at biopsy	Focus score	Dry eye symptoms	Ocular involvement	Dry mouth symptoms	Salivary flow	Autoantibodies		
							SSA/Ro	SSB/La	ANA
Non-SS	29	2	Yes	Yes	N/A	N/A	Positive	Negative	Positive
Non-SS	56	0	No	Yes	Yes	0	Positive	Negative	Positive
Non-SS	65	0	Yes	Yes	Yes	0.7	Negative	Negative	N/A
Non-SS	67	0	No	N/A	Yes	1.2	Negative	Negative	Negative
Non-SS	70	0	Yes	Yes	Yes	0.4	Negative	Negative	Negative
Non-SS	46	0	Yes	No	Yes	0.2	Negative	Negative	Negative
Non-SS	46	0	Yes	Yes	No	0.4	Negative	Negative	Positive
Non-SS	56	0	Yes	Yes	Yes	0.1	Negative	Negative	N/A
Non-SS	37	1	Yes	Yes	Yes	N/A	Negative	Negative	Negative
Non-SS	58	0	No	Yes	Yes	0.1	Negative	Negative	Negative
Non-SS	37	1	Yes	Yes	Yes	0.3	Negative	Negative	Negative
Non-SS	34	1	Yes	Yes	No	0.3	Negative	Negative	Negative
Non-SS	58	0	Yes	Yes	Yes	0	Negative	Negative	Negative
Non-SS	26	0	Yes	Yes	No	0.1	Negative	Negative	Negative
Non-SS	33	0	No	Yes	No	0.3	Negative	Negative	Negative
Non-SS	41	0	No	Yes	No	1.1	Positive	Negative	Negative
Non-SS	53	0	Yes	Yes	Yes	0.1	Negative	Negative	Negative
Non-SS	59	0	Yes	Yes	No	0.1	Negative	Negative	Negative
Non-SS	53	1	Yes	Yes	Yes	0.2	Negative	Negative	Negative
Non-SS	33	2	No	No	No	0.4	Positive	Positive	Negative
Non-SS	65	1	No	No	No	0.6	Negative	Negative	Negative
Non-SS	68	0	Yes	Yes	Yes	0.1	Negative	Negative	Negative
Non-SS	69	0	Yes	Yes	Yes	N/A	Negative	N/A	Negative
Non-SS	77	1	Yes	Yes	No	0	Negative	Negative	Negative
Non-SS	53	1	Yes	Yes	Yes	0.3	Negative	Negative	Negative
Non-SS	71	1	Yes	Yes	Yes	N/A	Negative	Negative	Negative
Non-SS	50	0	Yes	Yes	Yes	N/A	Negative	Negative	Negative
Non-SS	48	1	Yes	Yes	Yes	0.1	Negative	Negative	Positive
Non-SS	59	1	No	Yes	Yes	0	Negative	Negative	Negative
Primary SS	51	4	Yes	Yes	Yes	0.1	Positive	Positive	Negative
Primary SS	48	2	Yes	Yes	Yes	0.2	Positive	Positive	N/A
Primary SS	58	4	Yes	Yes	Yes	0.2	Negative	Negative	Negative
Primary SS	29	4	Yes	Yes	Yes	0.2	Positive	Negative	Positive
Primary SS	51	4	Yes	Yes	No	0.1	Positive	Positive	Negative
Primary SS	49	3	Yes	Yes	Yes	0.1	Positive	Positive	Positive
Primary SS	43	1	Yes	Yes	Yes	0.1	Negative	Negative	Negative
Primary SS	43	3	Yes	Yes	Yes	0.5	Negative	Negative	Negative
Primary SS	37	4	Yes	Yes	Yes	0.2	Positive	Positive	Positive
Primary SS	69	4	Yes	Yes	Yes	0.1	Negative	Negative	Negative
Primary SS	62	4	Yes	No	Yes	N/A	Positive	Positive	Positive
Primary SS	66	3	Yes	Yes	Yes	0	Positive	Positive	Positive
Primary SS	59	1	Yes	Yes	Yes	N/A	Negative	Negative	Negative
Primary SS	51	4	Yes	Yes	Yes	N/A	Positive	Positive	Negative
Primary SS	56	1	Yes	Yes	Yes	0.2	Positive	Negative	Negative
Primary SS	36	2	Yes	Yes	Yes	0.1	Positive	Positive	Positive
Primary SS	50	1	Yes	Yes	Yes	0.1	Positive	Positive	Negative
Primary SS	54	2	No	Yes	Yes	0.1	Negative	Negative	Positive
Primary SS	56	2	Yes	Yes	No	0.1	Positive	Positive	Positive
Primary SS	41	4	Yes	Yes	Yes	0	Positive	Positive	Positive
Primary SS	35	4	Yes	Yes	Yes	0	Positive	Positive	Positive
Primary SS	51	0	Yes	Yes	Yes	0.2	Positive	Positive	Negative
Primary SS	75	4	Yes	Yes	Yes	0	Negative	Negative	Positive
Primary SS	68	4	Yes	Yes	Yes	0.4	Positive	Positive	Positive
Primary SS	56	2	Yes	Yes	Yes	0.1	Positive	Negative	Negative
Primary SS	72	4	Yes	Yes	Yes	0	Negative	Negative	Positive
Primary SS	40	4	Yes	Yes	Yes	0.2	Positive	Positive	Positive
Primary SS	51	4	Yes	Yes	Yes	0.6	Positive	Positive	Negative
Primary SS	69	3	Yes	Yes	Yes	0.1	Positive	Positive	Positive
Primary SS	22	4	No	No	No	0.1	Positive	Negative	Positive
Primary SS	80	3	Yes	Yes	Yes	0	Negative	Negative	Positive
Primary SS	41	4	No	No	No	0.1	Positive	Negative	Positive
Primary SS	40	4	Yes	Yes	Yes	0.1	Positive	Positive	Positive
Primary SS	49	4	Yes	Yes	Yes	0.4	Positive	Negative	Negative
Primary SS	39	4	Yes	Yes	Yes	0	Positive	Positive	Positive
Primary SS	68	3	Yes	Yes	Yes	0.1	Positive	Negative	Negative
Primary SS	65	4	Yes	Yes	Yes	0.1	Positive	Positive	Positive
Primary SS	27	3	Yes	Yes	No	0.3	Positive	Positive	Positive
Primary SS	76	4	Yes	Yes	Yes	0.1	Negative	Negative	Negative
Primary SS	53	1	Yes	Yes	Yes	0.1	Negative	Negative	Negative
Primary SS	55	0	Yes	Yes	Yes	0.1	Positive	Negative	Positive
Primary SS	33	2	Yes	Yes	Yes	0.1	Negative	Negative	Negative
Primary SS	67	1	No	Yes	Yes	0	Negative	Negative	Negative

### Cells and plasmids

HSG cells were provided by Dr. Indu Ambudkar and were cultured in DMEM (ThermoFisher Scientific, Waltham, MA, USA) supplemented with 10% FBS at 37 ℃ in 5% CO_2_. HSG cells, which based on short tandem repeat analysis share a common origin with HeLa cells, have been used for a model for studying the molecular mechanisms of salivary cells. A253 cells (a cell line of salivary gland origin) were purchased from ATCC (Manassas, VA, USA) and cultured in McCoy’5A Medium (ThermoFisher Scientific, Waltham, MA, USA) supplemented with 10% FBS at 37 ℃ in 5% CO_2_. NS-SV-AC cells were donated by Professor M. Azuma and were cultured in Defined Keratinocyte SFM (ThermoFisher Scientific, Waltham, MA, USA) at 37 ℃ in 5% CO_2_^53^. These cells were confirmed as mycoplasma free by using MycoAlert (Lonza, Allendale, NJ, USA). pME18S-empty and pME18S-LAMP3 plasmids were prepared by cloning the LAMP3 open reading frame into pME18S expression vector containing a Kozak consensus sequence^54^. The full nucleotide sequences were confirmed by sequencing. pCMV6-LAMP1 plasmid was purchased from OriGene (SC116652; Rockville, MD, USA). These plasmids were purified using Endofree plasmid maxi kit (QIAGEN, Valencia, CA, USA).

### Establishment and culture of primary salivary gland cells

MSG biopsies was obtained from the consenting healthy volunteers and Sjögren syndrome patients (pSS) in the NIDCR Sjögren’s Syndrome Clinic. A table of patient information is provided in Supplementary Table S4. Human primary salivary gland epithelial cells (pSGECs) were isolated and grown on collagen-coated plates (Biocoat, Becton Dickinson) as previously described^55^. Briefly, pSGECs were maintained in complete KGM2 (Lonza, Allendale, NJ, USA) supplemented with bovine pituitary extracts (BPE), recombinant human epidermal growth factor (hEGF), insulin (INS), hydrocortisone (HC), gentamicin, epinephrine and transferrin and the calcium concentration was adjusted to 0.05 mM with CaCl_2_ solution. To promote the differentiation of pSGECs, the calcium concentration in the completed KGM2 was adjusted to 1.2 mM and the pSGECs cultures were maintained for 3 days before the extraction of total RNA.

### Measurement of LAMP3 gene expression in culture of human pSGECs

*LAMP3* mRNA expression was measured using quantitative real-time polymerase chain reaction and Taqman primer sets. Briefly, equal amount of total RNAs of each pSGECs were first reverse-transcribed into cDNA using iScript supermix (BioRad, Hercules, CA, USA). cDNAs were amplified for *ACTB* (Hs01060665_g1) and *LAMP3* (Hs01111316_m1) and data was collected using a StepOnePlus (Applied Biosystems). *ACTB* was used as an internal control for normalization of input cDNA, and the difference of the cycle threshold (Ct) *LAMP3* was calculated using the *Δ*Ct method and used to determine the relative quantitation (RQ) values (2^−*ΔΔ*Ct^), which represent the relative level of fold change to control.

### Transient transfection

HSG and A253 cells (5 × 10^5^ cells) were transfected with a total amount of 1.5 μg pME18S-empty and pME18S-LAMP3 plasmid using Lipofectamine 3000 (ThermoFisher Scientific, Waltham, MA, USA). These cells were used for Western blotting with anti-caspase3 pAb 72 h after transfection and used for the other experiments 48 h after of transfection.

### Isolation of extracellular vesicles

Twenty-four hours after transfection, cells were washed in PBS twice, and then 2.5 × 10^6^ cells of transfected cells were re-plated on 10-cm dish with 10 ml of media supplied with 10% exosome-depleted FBS (Thermofisher Scientific, Waltham, MA, USA) and anti-biotic anti-mycotic. After 72 h, culture media was collected, and centrifuged at 2000*g* for 30 min at 4 ℃ to eliminate cells and debris. The supernatant was incubated with total exosome isolation reagent overnight at 4 ℃ (Thermofisher Scientific, Waltham, MA, USA). The mixture was centrifuged at 10,000*g* for 60 min at 4 ℃. The pellets contained extracellular vesicles (EVs), including apoptotic bodies, were used for Western blotting as described in “Supplementary Methods”. Immunofluorescent labeling, confocal imaging, and protein expression quantification are also detailed in “Supplementary Methods”.

### Cell growth and cell cycle analysis

Five × 10^5^ HSG and A253 cells were transfected with 1.5 μg pME18S-empty or 1.5 μg pME18S-LAMP3 plasmid. Twenty-four hours after transfection, cells were re-plated at 2 × 10^5^ cells in 10-cm culture dish. Four days after re-plating, cells were counted by using trypan blue solution and Countess Automated Cell Counter (Thermofisher Scientific, Waltham, MA, USA). For cell cycle analysis, 1 × 10^6^ cells were fixed and permeabilized with 80% etanohol five days after re-plating, and then incubated in 80% etanohol for 24 h at 4 ℃. Cells were washed once in cold-PBS, and then washed once in cold-PBS supplied with 2% FBS. After washing, cells were treated with PI/RNase Staining Buffer (550825, BD Bioscience, San Jose, CA, USA) for 15 min at 25 ℃. Stained cells were analyzed by using the BD Accuri (BD Biosciences, San Jose, CA, USA) using the BD CSampler software.

### Apoptosis assay

Five × 10^5^ HSG and A253 cells were transfected with a total amount of 1.5 μg pME18S-empty or pME18S-LAMP3 plasmid, and 5 × 10^5^ HSG and A253 cells were co-transfected with 0.75 μg AAV2-GFP and 0.75 μg in pME18S-empty, pME18S-LAMP3, pUC or pCMV-LAMP1 plasmid using Lipofectamine 3000. Twenty-four hours after transfection, the cells were re-plated at 3 × 10^5^ cells per well in 6-well plate, then 24- or 48-h after re-plating, cells were used to detect apoptotic cells. To examine whether the apoptosis induced by LAMP3 was via activated caspase, cells were treated with or without 20 μM Z-VAD-FMK (Z-VAD) 24 h after transfection. After incubation for 20 h, cells were trypsinized and used to detect apoptotic cells by flow cytometry using the APC Annexin V Apoptosis Detection Kit with 7-AAD (640930, Biolegend, San Diego, CA, USA) and the BD Accuri (BD Biosciences, San Jose, CA, USA) using the BD CSampler software.

### Statistical analysis

Statistical analysis of microarray studies was performed as described above. Two-tailed Student's *t*- or analysis of variance (ANoVA) tests, where appropriate, were employed as indicated using JMP 13.2.0 (SAS Institute, Cary, NC). P-values less than 0.05 were considered statistically significant. Sample size calculations were based on an independent two-sample *t-*test with unequal variances to show that group sizes of at least 16 would be sufficient to have at least 80% power to detect differences where the difference in the means was at least twofold and the standard deviation equal to the difference in the means at a significant 5% level.

### Human subjects research declaration

All clinical investigations were conducted in accordance with the Declaration of Helsinki principles. Written informed consent to IRB-approved protocols (as described above) were obtained from all participants prior to inclusion to the studies described herein. All human studies were approved by the appropriate institutional review boards at each site where tissues were procured.

## Supplementary information

Supplementary Information.
