# Supplementary material for: LAMP3 induces apoptosis and autoantigen release in Sjögren’s syndrome patients
Source: Sci Rep. 2020 Sep 16;10:15169. doi: 10.1038/s41598-020-71669-5 (PMC7494869; doi:10.1038/s41598-020-71669-5)
Supplement: Supplementary file 1 — Supplementary Information. [file 41598_2020_71669_MOESM1_ESM.pdf]

Supplementary Information
Scientific Reports

LAMP3 induces apoptosis and autoantigen release in Sjögren's syndrome patients

Tsutomu Tanaka[1*], Blake M. Warner[1*], Toshio Odani[1], Youngmi Ji[1], Ying-Qian Mo[1], Hiroyuki Nakamura[1], Shyh-Ing Jang[1], Hongen Yin[1], Drew G. Michael[1], Noriyuki Hirata[2], Futoshi Suizu[2], Satoko Ishigaki[2], Fabiola Reis Oliveira[3], Ana Carolina F. Motta[4], Alfredo Ribeiro-Silva[5], Eduardo M. Rocha[6], Tatsuya Atsumi[7], Masayuki Noguchi[2], John A. Chiorini[1+]

1. National Institute of Dental and Craniofacial Research, National Institutes of Health, Bethesda, MD, USA.
2. Division of Cancer Biology, Institute for Genetic Medicine, Hokkaido University, Sapporo, Japan
3. Department of Clinical Medicine, Ribeirão Preto Medical School, University of Sao Paulo, Ribeirao Preto, Brazil
4. Department of Stomatology, Public Health and Forensic Dentistry, School of Dentistry of Ribeirao Preto, University of Sao Paulo, Brazil
5. Department of Pathology and Legal Medicine, Ribeirao Preto Medical School, University of Sao Paulo, Ribeirao Preto, SP, Brazil
6. Department of Ophthalmology, Otorhinolaryngology, Head and Neck Surgery, Ribeirão Preto Medical School, University of Sao Paulo, Ribeirao Preto, Brazil
7. Department of Rheumatology, Endocrinology and Nephrology, Faculty of Medicine and Graduate School of Medicine, Hokkaido University, Sapporo, Japan

* - these authors contributed equally to this work.

[+]Author to whom correspondence should be addressed
John A. Chiorini
AAV Biology Section
NIH 10 Center Dr.
Bethesda MD 20892
Phone 301-496-4279
Fax 301-402-1228
Email: jchiorini@dir.nidcr.nih.gov

Supplementary Materials and Methods

Transient transfection

One μg of pME18S-LAMP3 plasmid or pME18S-empty plasmid as control was introduced to NS-SV-AC cells by using Amaxa Nucleofector and Amaxa Cell Line Nucleofector Kit L. Forty-eight hours after transfection, cells were used for apoptosis assay.

RNA isolation and qRT-PCR analysis

Total RNA from human subjects was extracted using the RNeasy Mini Kit following the manufacturer's recommended protocol (Qiagen, MD, USA). Extracted RNA was treated with TURBO DNase (ThermoFisher Scientific, MD, USA). The concentration and quality of total RNA was measured using NanoDrop (Thermo Fisher Scientific, MD, USA). For qRT-PCR, total RNA was reverse transcribed into cDNA using the High-Capacity RNA-to-cDNA Kit (Thermo Fisher Scientific, MD, USA); 5 ng of cDNA was used as qPCR template in triplicate. TaqMan Gene Expression Assays: *LAMP3* (Hs01111316_m1) and *ACTB* (Hs01060665_g1) were used to measure gene expression (Thermo Fisher Scientific, MD, USA). Relative gene expression was calculated using the $2^{-\Delta\Delta Ct}$ method (1). Data was analyzed by using GraphPad PRISM 8.0 software and presented as mean ± s.e.m. For statistical analysis, P values were calculated by using the unpaired, two-tailed Student's *t*-test. P values were considered statistically significant if $P< 0.05$.

qRT-PCR analysis

Total RNA was isolated from cultured cells using RNeasy Mini kits (QIAGEN, Valencia, CA) according to the manufacturer's instructions. cDNA was synthesized from 1μg total RNA using Superscript III reverse transcriptase (RT) and oligo (dT) primer (Thermo Fisher Scientific, MD) according to the manufacturer's protocol. Quantitative real-time RT polymerase chain reaction (qRT-PCR) was performed to determine the mRNA expression of *CASP3*

(Hs00234387_m1; Thermo Fisher Scientific, MD) relative to *ACTB* using the *ΔΔCt* method (1). Fold-change in expression of the indicated genes represents the mean (±SD) of triplicate reactions. PCR cycles were performed on the Quantstudio3 Real-Time PCR System (Life Technologies) with the following cycle conditions: 2 minutes at 50°C, 10 minutes at 95°C, 50 cycles of 15 s at 95°C and 1 minute at 60°C.

Western blotting

Cultured cells were washed in ice-cold PBS, lysed by incubation for 30 minutes on ice in RIPA buffer supplemented with protease inhibitor, phosphatase inhibitor (Thermo Fisher Scientific, MD), and 25µM CQ or 20µM pepA, and cleared by centrifugation at 17,000 g for 25 minutes at 4°C. Supernatants and EVs isolated from culture media were heated for 10 minutes at 97°C in LDS sample buffer, resolved by SDS-PAGE, and electrophoretically transferred to PVDF membranes (Invitrolon™ PVDF; Thermo Fisher Scientific, MD). The membranes were incubated with blocking buffer (5% non-fat dried milk or Bovine Serum Albumin (Sigma-Aldrich, St. Louis, MO, USA) in wash buffer (0.1% Tween-20 in TBS)) for 60 minutes at 25°C then incubated overnight with primary antibodies: anti-caspase-3 pAb (9662; Cell Signaling Technology Danvers, MA, USA) and Flotillin-1 mAb (clone:D2V7J; 18634S; Cell Signaling Technology); Anti-LAMP3 pAb (12632-1-AP; Proteintech, Inc., Rosemont, IL, USA), Anti-SSB mAb (clone: mAbcam75927; ab75927; Abcam, Cambridge, MA); Anti-ATF4 mAb (clone: S360A-24; NBP2-42176; Novus Biologicals, Littleton, CO, USA) and anti-XBP1 pAb (NBP1-77681; Novus Biologicals, Littleton, CO); Anti-52kDa Ro/SSA (clone: D-12; sc-25351; Santa Cruz Biotechnology, Dallas, TX, USA); Anti-α-tubulin mAb (clone: DM1A, Sigma-Aldrich, St Louis MO). After washing three times with wash buffer (0.1% Tween-20 in TBS), the membranes were reacted with Mouse IgG HRP-linked whole antibody (GENA931; Sigma-Aldrich, St Louis MO)

or Rabbit IgG HRP-linked whole antibody (GENA934; Sigma-Aldrich, St Louis MO) for 1 hour at 25°C. Signal was visualized using a Super Signal West Pico Chemiluminescent Substrate or Super Signal West Pico PLUS Chemiluminescent Substrate (Thermo Fisher Scientific, MD) according to the manufacturer's protocol.

Immunofluorescent labeling and protein expression quantification

Tissue sections (5μm) were cut from formalin-fixed paraffin embedded (FFPE) minor salivary gland (MSG) biopsy blocks. FFPE sections were deparaffinized, rehydrated, and subjected to citric acid microwave antigen retrieval. Slides were then blocked with 5% donkey serum (Jackson ImmunoResearch, West Grove, PA) and 0.5% BSA (Sigma Aldrich Corp, The Woodlands, TX) in PBS for 1 hour at 25°C in a humidified chamber then incubated at 4°C overnight with 100μl of 10 μg/mL primary antibodies as listed: LAMP3 (12632-1-AP, Proteintech, Rosemont IL). Slides were then washed in 5 changes of PBS for 5 minutes each, then incubated with 1:200 dilution of 2 mg/mL AlexaFluor 488 donkey anti-rabbit IgG (Cat. 711-545-152, Jackson ImmunoResearch, West Grove, PA) secondary antibody for 1 hour at 25°C in the dark, followed by washing in 5 changes of PBS for 5 minutes each, and counterstaining with DAPI mounting medium. All images were acquired by using Olympus Fluoview 1000 software. Analysis and quantification of expression were performed by using Icy v.1.9.10.0 (BioImage Analysis unit Institut Pasteur, Paris, France).

Confocal microscopy

Cultured cells were washed with PBS three times, and then fixed with 4% paraformaldehyde for 10 minutes then washed three times with PBS. Cells were permeabilized with 100% -20°C methanol for 10 minutes and 0.5% saponin in PBS for 10 minutes. Cells were then washed with PBS five times. Cells were incubated with blocking buffer (0.5%

immunoglobulin-free BSA, 0.1% saponin, 2.2% glycine, 0.1% Tween-20 and 5% donkey serum in PBS). Cells were then incubated overnight with antibodies: Anti-52kDa Ro/SSA mAb (Santa Cruz Biotechnology; clone: D-12; sc-25351) or anti-SSB mAb (Abcam; clone: mAbcam75927; ab75927). After washing with PBS five times, cells were incubated with 2 mg/mL AlexaFluor 594 donkey anti-mouse IgG secondary antibody (Cat. 711-585-152; Jackson ImmunoResearch Laboratories, Inc., West Grove, PA, USA) at 25℃. Cells were then washed five times in PBS and then mounted. Areas with cells were imaged using an Olympus confocal microscope with silicone immersion 60X or 100X objective with 22 stacked sections at a z-dimension of 0.50 or 0.30 μm per section, respectively. Image analysis was performed using Icy v.1.9.10.0 applications.

Reference

1. Pfaffl MW. A new mathematical model for relative quantification in real-time RT-PCR. *Nucleic Acids Res.* 2001; 29(9): e45

Supplementary Table S1. Two hundred and seventy-nine common genes with significantly differential expression (all $p<0.05$) in minor salivary glands among four comparisons from 3 transcriptome studies

Gene Symbol	Gene Name	Comparison #1 pSS (n=8) vs HV (n=6) Yin, H., 2013 [1]		Comparison #2 pSS (n=10) vs non-SS (n=5) Greenwell-Wild, T., 2011 [2]		Comparison #3 pSS (n=3) vs HV (n=3) Tsuboi, H., 2014 [3]		Comparison #4 pSS (n=3) vs IgG4RD (n=5) Tsuboi, H., 2014 [3]		Average of fold change (FC)
		FC	*p* value	FC	*p* value	FC	*p* value	FC	*p* value	
CXCL11	chemokine (C-X-C motif) ligand 11	5.891	0.015	4.827	0.001	4.206	0.039	4.966	0.021	4.972
IFI44L	interferon-induced protein 44-like	5.287	0.007	4.710	0.004	5.009	0.002	3.695	0.001	4.675
IFI44	interferon-induced protein 44	5.116	0.002	3.513	0.001	5.765	0.002	2.560	<0.001	4.238
XAF1	XIAP associated factor 1	5.595	0.001	5.140	<0.001	3.120	0.011	2.380	0.001	4.059
STAT1	signal transducer and activator of transcription 1	3.882	0.010	4.253	<0.001	4.807	0.003	3.143	0.009	4.021
RSAD2	radical S-adenosyl methionine domain containing 2	5.618	0.001	2.868	0.007	4.434	0.004	2.618	0.002	3.885
**LAMP3**	**lysosomal-associated membrane protein 3**	**5.177**	**<0.001**	**3.785**	**<0.001**	**2.621**	**0.023**	**3.484**	**0.012**	**3.767**
RGS1	regulator of G-protein signaling 1	4.309	<0.001	3.065	0.003	2.635	0.038	4.496	0.006	3.626
EPSTI1	epithelial stromal interaction 1 (breast)	5.535	0.003	3.106	<0.001	2.857	0.008	2.540	0.001	3.510
KIAA0125	KIAA0125	5.185	<0.001	2.715	0.005	2.849	0.024	2.772	0.044	3.380
SAMD9L	sterile alpha motif domain containing 9-like	5.283	0.001	3.112	<0.001	2.965	0.020	2.129	0.017	3.372
DDX60	DEAD (Asp-Glu-Ala-Asp) box polypeptide 60	4.600	0.004	3.550	<0.001	3.312	0.005	1.807	0.008	3.317
IFIT3	interferon-induced protein with tetratricopeptide repeats 3	3.682	0.024	3.801	0.001	2.867	0.012	1.859	0.023	3.052
SLAMF7	SLAM family member 7	3.986	0.004	1.938	0.024	2.571	0.040	3.614	0.016	3.028
PKHD1L1	polycystic kidney and hepatic disease 1 (autosomal recessive)-like 1	5.044	0.003	1.854	0.029	2.274	0.035	2.443	0.019	2.904
TLR7	toll-like receptor 7	3.937	0.008	2.909	0.017	2.513	0.018	1.762	0.047	2.780
IFI27	interferon, alpha-inducible protein 27	4.416	0.004	2.343	0.015	2.260	0.009	1.904	0.027	2.731
MANEA	mannosidase, endo-alpha	4.061	0.006	1.647	0.001	3.181	0.002	1.961	0.025	2.713
ZNF638	zinc finger protein 638	2.005	0.024	5.183	0.001	2.169	0.003	1.316	0.001	2.668
PARP9	poly (ADP-ribose) polymerase family member 9	3.583	0.003	2.984	<0.001	2.170	0.006	1.858	0.004	2.649
IFIT1	interferon-induced protein with tetratricopeptide repeats 1	3.634	0.006	2.102	0.025	2.326	0.006	2.133	0.002	2.549

Gene Symbol	Gene Name	Comparison #1 pSS (n=8) vs HV (n=6) Yin, H., 2013 [1]		Comparison #2 pSS (n=10) vs non-SS (n=5) Greenwell-Wild, T., 2011 [2]		Comparison #3 pSS (n=3) vs HV (n=3) Tsuboi, H., 2014 [3]		Comparison #4 pSS (n=3) vs IgG4RD (n=5) Tsuboi, H., 2014 [3]		Average of fold change (FC)
		FC	*p* value	FC	*p* value	FC	*p* value	FC	*p* value	
VPS13C	vacuolar protein sorting 13 homolog C (S. cerevisiae)	2.012	0.022	5.191	<0.001	1.688	0.018	1.176	0.030	2.516
HSPA13	heat shock protein 70kDa family, member 13	2.933	0.005	2.341	<0.001	2.171	0.002	1.943	0.011	2.347
PARP14	Poly (ADP-ribose) polymerase family member 14	3.871	0.001	1.698	0.009	2.067	0.017	1.533	0.040	2.292
SUB1	SUB1 homolog, transcriptional regulator	4.240	0.009	1.736	<0.001	1.562	0.022	1.366	0.020	2.226
DDX58	DEAD (Asp-Glu-Ala-Asp) box polypeptide 58	3.006	0.007	2.480	0.001	1.856	0.013	1.516	0.038	2.215
RAB30	RAB30, member RAS oncogene family	2.096	0.027	2.404	<0.001	2.567	0.023	1.791	0.020	2.215
OAS2	2'-5'-oligoadenylate synthetase 2	2.327	0.041	1.487	0.044	2.114	0.006	2.805	0.004	2.183
DTX3L	deltex 3 like, E3 ubiquitin ligase	2.595	0.008	2.515	<0.001	2.100	0.008	1.439	0.019	2.162
PDK1	pyruvate dehydrogenase kinase, isozyme 1	2.764	0.002	1.637	0.011	2.207	0.025	2.022	0.006	2.158
HERC6	HECT and RLD domain containing E3 ubiquitin protein ligase family member 6	2.128	0.035	1.917	0.035	2.153	0.015	2.363	<0.001	2.140
IFIT5	interferon-induced protein with tetratricopeptide repeats 5	2.951	0.001	1.856	0.001	2.269	0.002	1.418	0.013	2.124
C12orf4	chromosome 12 open reading frame 4	2.973	0.006	2.090	0.002	1.877	0.003	1.522	0.007	2.116
GLCCI1	glucocorticoid induced 1	2.316	0.008	1.843	0.003	2.481	0.003	1.740	0.033	2.095
DENND1B	DENN/MADD domain containing 1B	2.456	0.022	2.327	0.030	2.094	0.003	1.487	0.005	2.091
ETV7	ets variant 7	3.241	0.026	1.426	0.002	1.811	0.043	1.879	0.022	2.089
IFIH1	interferon induced, with helicase C domain 1	2.645	0.010	1.822	<0.001	2.270	0.017	1.468	0.007	2.052
UFM1	ubiquitin-fold modifier 1	2.941	0.003	2.143	0.006	1.625	0.012	1.405	0.014	2.029
TLR3	toll-like receptor 3	2.495	0.022	1.954	0.017	2.277	0.016	1.386	0.012	2.028
SMG1	SMG1 phosphatidylinositol 3-kinase-related kinase	2.072	0.031	2.764	<0.001	1.821	0.021	1.362	0.030	2.005
HERC5	HECT and RLD domain containing E3 ubiquitin protein ligase 5	2.151	0.027	1.880	0.010	2.067	0.011	1.920	<0.001	2.005
RNF213	ring finger protein 213	1.923	0.044	2.761	0.012	1.849	0.007	1.462	0.046	1.999

Gene Symbol	Gene Name	Comparison #1 pSS (n=8) vs HV (n=6) Yin, H., 2013 [1]		Comparison #2 pSS (n=10) vs non-SS (n=5) Greenwell-Wild, T., 2011 [2]		Comparison #3 pSS (n=3) vs HV (n=3) Tsuboi, H., 2014 [3]		Comparison #4 pSS (n=3) vs IgG4RD (n=5) Tsuboi, H., 2014 [3]		Average of fold change (FC)
		FC	*p* value	FC	*p* value	FC	*p* value	FC	*p* value	
ATM	ATM serine/threonine kinase	2.192	0.019	2.696	0.001	1.761	0.014	1.288	0.049	1.984
SLFN5	schlafen family member 5	1.665	0.025	2.252	0.004	2.509	0.026	1.390	0.015	1.954
SEL1L	sel-1 suppressor of lin-12-like (C. elegans)	1.830	0.035	1.560	0.009	2.406	0.023	1.814	0.011	1.902
ZNF280B	zinc finger protein 280B	3.245	0.029	1.545	0.003	1.430	0.033	1.288	0.021	1.877
HERC4	HECT and RLD domain containing E3 ubiquitin protein ligase 4	1.854	0.044	2.055	<0.001	2.442	0.019	1.154	0.037	1.876
SP100	SP100 nuclear antigen	2.024	0.007	2.085	0.003	1.902	0.015	1.425	0.026	1.859
C5orf56	chromosome 5 open reading frame 56	1.971	0.011	2.292	0.001	1.641	0.002	1.433	0.030	1.834
USP38	ubiquitin specific peptidase 38	2.590	0.003	1.518	0.009	1.962	0.008	1.190	0.040	1.815
BMP6	bone morphogenetic protein 6	3.143	0.005	-1.208	0.030	2.327	0.010	2.967	<0.001	1.807
SEC24A	SEC24 homolog A, COPII coat complex component	1.979	0.031	1.635	0.001	1.952	0.003	1.649	0.005	1.803
PPM1K	protein phosphatase, Mg2+/Mn2+ dependent, 1K	1.851	0.036	1.837	0.009	1.872	0.004	1.618	0.007	1.795
SLC44A1	solute carrier family 44 (choline transporter), member 1	1.776	0.042	1.745	0.010	1.993	0.014	1.653	0.022	1.792
LARP1B	La ribonucleoprotein domain family, member 1B	1.838	0.036	1.407	0.022	1.942	0.013	1.868	0.002	1.764
IFNAR1	interferon (alpha, beta and omega) receptor 1	2.324	0.017	1.461	0.001	1.742	0.034	1.522	0.001	1.762
LRIF1	ligand dependent nuclear receptor interacting factor 1	2.472	0.007	1.509	0.030	1.836	<0.001	1.204	0.035	1.755
ITCH	itchy E3 ubiquitin protein ligase	1.712	0.036	1.829	0.016	2.047	0.015	1.325	0.044	1.728
XIAP	X-linked inhibitor of apoptosis, E3 ubiquitin protein ligase	2.065	0.022	1.497	0.023	2.072	0.030	1.243	0.030	1.719
SLC33A1	solute carrier family 33 (acetyl-CoA transporter), member 1	1.754	0.031	1.534	0.027	1.881	0.004	1.678	0.002	1.712
SP140L	SP140 nuclear body protein-like	1.865	0.048	1.745	0.001	1.756	0.033	1.475	0.040	1.710
HS2ST1	heparan sulfate 2-O-sulfotransferase 1	1.963	0.027	1.508	0.002	1.980	0.001	1.370	0.026	1.705
FBXW7	F-box and WD repeat domain containing 7, E3 ubiquitin protein ligase	2.143	0.014	1.577	0.006	1.697	0.014	1.395	0.010	1.703

Gene Symbol	Gene Name	Comparison #1 pSS (n=8) vs HV (n=6) Yin, H., 2013 [1]		Comparison #2 pSS (n=10) vs non-SS (n=5) Greenwell-Wild, T., 2011 [2]		Comparison #3 pSS (n=3) vs HV (n=3) Tsuboi, H., 2014 [3]		Comparison #4 pSS (n=3) vs IgG4RD (n=5) Tsuboi, H., 2014 [3]		Average of fold change (FC)
		FC	*p* value	FC	*p* value	FC	*p* value	FC	*p* value	
DCUN1D1	DCN1, defective in cullin neddylation 1, domain containing 1	2.186	0.014	1.418	0.016	1.906	0.013	1.297	0.007	1.702
HLA-C	major histocompatibility complex, class I, C	1.792	0.038	1.490	0.018	1.686	0.022	1.805	0.010	1.693
ORMDL1	ORMDL sphingolipid biosynthesis regulator 1	1.827	0.026	1.575	0.018	2.005	0.011	1.325	0.003	1.683
PPHLN1	periphilin 1	1.596	0.039	1.808	0.007	1.880	0.003	1.427	0.002	1.678
TFAM	transcription factor A, mitochondrial	1.905	0.033	1.943	<0.001	1.652	0.010	1.149	0.044	1.662
RORA	RAR-related orphan receptor A	1.898	0.034	1.753	0.002	1.677	0.039	1.317	0.031	1.661
SPAG9	sperm associated antigen 9	1.781	0.033	1.757	0.014	1.695	0.046	1.396	0.004	1.657
TMEM19	transmembrane protein 19	1.919	0.047	1.603	0.006	1.549	0.040	1.499	0.015	1.643
MAN1A2	mannosidase, alpha, class 1A, member 2	1.853	0.043	1.285	0.044	1.952	0.006	1.435	0.036	1.631
ZBTB38	zinc finger and BTB domain containing 38	1.805	0.046	1.445	0.006	1.952	0.004	1.301	0.008	1.626
TIPARP	TCDD-inducible poly (ADP-ribose) polymerase	2.180	0.012	1.514	<0.001	1.418	0.004	1.362	0.005	1.618
RINT1	RAD50 interactor 1	1.938	0.010	1.617	<0.001	1.690	0.006	1.215	0.047	1.615
NGLY1	N-glycanase 1	2.023	0.021	1.697	0.028	1.366	0.038	1.334	0.024	1.605
USP48	ubiquitin specific peptidase 48	1.918	0.029	1.590	<0.001	1.457	0.018	1.400	<0.001	1.591
XRCC4	X-ray repair complementing defective repair in Chinese hamster cells 4	2.403	0.025	1.368	0.036	1.398	0.003	1.194	0.022	1.591
SLC25A46	solute carrier family 25, member 46	1.794	0.039	1.692	<0.001	1.666	0.024	1.200	0.045	1.588
LOC645513	uncharacterized LOC645513	1.943	0.021	1.600	0.024	1.426	0.017	1.324	0.020	1.573
IQCB1	IQ motif containing B1	1.780	0.033	1.580	0.006	1.516	0.015	1.369	0.005	1.561
TOR1AIP2	torsin A interacting protein 2	1.912	0.034	1.514	0.004	1.637	0.050	1.181	0.032	1.561
SYNRG	synergin, gamma	1.562	0.036	1.720	<0.001	1.530	0.028	1.411	0.007	1.556
DDI2	DNA-damage inducible 1 homolog 2	1.842	0.042	1.415	0.023	1.640	0.003	1.323	0.039	1.555
PSMA3	proteasome subunit alpha 3	1.871	0.042	1.392	0.003	1.685	0.017	1.245	0.020	1.548
MDFIC	MyoD family inhibitor domain containing	1.858	0.034	1.503	0.010	1.418	0.013	1.396	0.019	1.544
CEP57L1	centrosomal protein 57kDa-like 1	2.213	0.028	1.213	0.025	1.418	0.025	1.317	0.004	1.540

Gene Symbol	Gene Name	Comparison #1 pSS (n=8) vs HV (n=6) Yin, H., 2013 [1]		Comparison #2 pSS (n=10) vs non-SS (n=5) Greenwell-Wild, T., 2011 [2]		Comparison #3 pSS (n=3) vs HV (n=3) Tsuboi, H., 2014 [3]		Comparison #4 pSS (n=3) vs IgG4RD (n=5) Tsuboi, H., 2014 [3]		Average of fold change (FC)
		FC	*p* value	FC	*p* value	FC	*p* value	FC	*p* value	
BROX	BRO1 domain and CAAX motif containing	2.210	0.019	1.216	0.039	1.487	0.009	1.227	0.028	1.535
GIN1	gypsy retrotransposon integrase 1	2.025	0.035	1.483	0.019	1.349	0.034	1.243	0.028	1.525
KIAA1033	KIAA1033	3.089	0.005	1.651	<0.001	2.589	0.006	-1.260	0.016	1.518
UBA6	ubiquitin-like modifier activating enzyme 6	2.740	0.009	1.865	0.001	2.676	0.032	-1.240	0.004	1.510
PNPT1	polyribonucleotide nucleotidyltransferase 1	2.025	0.016	1.238	0.042	1.477	0.018	1.270	0.003	1.503
LYRM2	LYR motif containing 2	2.018	0.014	1.241	0.031	1.405	0.016	1.339	0.002	1.501
NUP50	nucleoporin 50kDa	1.850	0.021	1.372	0.002	1.495	0.014	1.279	0.028	1.499
TSEN15	TSEN15 tRNA splicing endonuclease subunit	1.959	0.026	1.460	0.008	1.288	0.042	1.289	0.026	1.499
LRRFIP1	leucine rich repeat (in FLII) interacting protein 1	2.428	0.049	3.605	0.002	1.503	0.025	-1.579	0.009	1.489
ELMOD2	ELMO/CED-12 domain containing 2	1.922	0.022	1.406	0.004	1.323	0.041	1.304	0.016	1.489
PPP1R2	protein phosphatase 1, regulatory (inhibitor) subunit 2	1.585	0.046	1.537	0.017	1.553	0.003	1.196	0.015	1.468
QKI	QKI, KH domain containing, RNA binding	2.503	0.029	3.172	0.002	1.776	0.036	-1.583	0.017	1.467
MBNL1	muscleblind like splicing regulator 1	2.123	0.011	2.112	<0.001	2.970	0.014	-1.398	0.020	1.452
PSMD12	proteasome 26S subunit, non-ATPase 12	1.726	0.048	1.344	0.009	1.396	0.011	1.266	0.017	1.433
NSUN3	NOP2/Sun domain family, member 3	1.930	0.043	1.254	0.041	1.331	0.037	1.206	0.029	1.430
CLDND1	claudin domain containing 1	1.788	0.049	1.409	0.002	1.269	0.038	1.169	0.020	1.409
REL	v-rel avian reticuloendotheliosis viral oncogene homolog	2.094	0.013	2.311	0.001	2.320	0.014	-1.267	0.039	1.364
DNAJB14	DnaJ (Hsp40) homolog, subfamily B, member 14	2.535	0.010	1.863	0.001	2.137	0.002	-1.239	0.004	1.324
SMC4	structural maintenance of chromosomes 4	2.323	0.018	2.207	<0.001	2.013	0.022	-1.287	0.016	1.314
ACAP2		2.401	0.009	2.115	<0.001	1.814	0.022	-1.284	0.005	1.261
MAN1A1	mannosidase, alpha, class 1A, member 1	2.460	0.039	1.735	0.017	2.126	0.030	-1.300	0.013	1.256
SP3	Sp3 transcription factor	2.026	0.027	2.421	0.008	1.760	0.009	-1.205	0.009	1.251

Gene Symbol	Gene Name	Comparison #1 pSS (n=8) vs HV (n=6) Yin, H., 2013 [1]		Comparison #2 pSS (n=10) vs non-SS (n=5) Greenwell-Wild, T., 2011 [2]		Comparison #3 pSS (n=3) vs HV (n=3) Tsuboi, H., 2014 [3]		Comparison #4 pSS (n=3) vs IgG4RD (n=5) Tsuboi, H., 2014 [3]		Average of fold change (FC)
		FC	*p* value	FC	*p* value	FC	*p* value	FC	*p* value	
RECQL	RecQ helicase-like	2.345	0.011	2.016	0.001	2.030	0.008	-1.392	0.003	1.250
DCLRE1C	DNA cross-link repair 1C	2.208	0.020	2.119	0.005	1.987	0.050	-1.344	0.041	1.243
ZEB2	zinc finger E-box binding homeobox 2	2.828	0.020	2.226	0.043	2.141	0.029	-2.233	0.001	1.241
CCDC82	coiled-coil domain containing 82	2.694	0.009	1.742	0.001	1.812	0.006	-1.331	0.003	1.229
IFI16	interferon, gamma-inducible protein 16	2.939	0.004	1.650	0.009	1.500	0.011	-1.218	0.043	1.218
CCDC88A	coiled-coil domain containing 88A	2.524	0.043	1.945	0.018	1.913	0.012	-1.616	0.002	1.191
EPS15	epidermal growth factor receptor pathway substrate 15	2.538	0.005	1.844	0.004	1.617	0.015	-1.282	0.009	1.179
IFT80	intraflagellar transport 80	1.932	0.045	2.124	0.001	1.777	0.008	-1.199	0.029	1.158
PTPN11	protein tyrosine phosphatase, non-receptor type 11	1.960	0.026	1.339	0.036	2.532	0.009	-1.209	0.011	1.156
FMR1	fragile X mental retardation 1	1.973	0.041	1.952	0.004	1.801	0.013	-1.209	0.035	1.129
MBTD1	mbt domain containing 1	1.756	0.049	1.575	0.049	2.333	0.021	-1.195	0.045	1.117
CEP290	centrosomal protein 290kDa	2.767	0.035	1.557	0.003	1.600	0.005	-1.456	0.015	1.117
MTR	5-methyltetrahydrofolate-homocysteine methyltransferase	1.969	0.019	2.268	0.001	1.530	0.032	-1.334	0.001	1.108
CBX3	chromobox homolog 3	1.872	0.042	1.916	0.010	1.813	0.021	-1.187	0.029	1.103
NR2C1	nuclear receptor subfamily 2, group C, member 1	1.868	0.010	2.349	0.001	1.707	0.007	-1.515	0.008	1.102
BLZF1	basic leucine zipper nuclear factor 1	2.623	0.025	1.434	0.005	1.668	0.009	-1.362	0.049	1.091
LRRC40	leucine rich repeat containing 40	2.183	0.016	1.892	0.003	1.616	0.008	-1.330	0.002	1.090
KIDINS220	kinase D-interacting substrate 220kDa	1.804	0.037	2.237	0.019	1.569	0.019	-1.258	0.033	1.088
NEK1	NIMA-related kinase 1	2.227	0.042	1.403	0.005	1.989	0.005	-1.291	0.024	1.082
LRR1	leucine rich repeat protein 1	2.629	0.011	1.569	0.002	-1.276	0.024	1.379	0.017	1.075
LUC7L3	LUC7-like 3 pre-mRNA splicing factor	1.812	0.044	2.331	<0.001	1.637	0.037	-1.484	0.003	1.074
FAM3C	family with sequence similarity 3, member C	1.739	0.050	1.620	0.005	2.108	0.008	-1.183	0.009	1.071
SUGT1	SGT1 homolog, MIS12 kinetochore complex assembly cochaperone	1.963	0.030	1.737	0.015	1.833	0.049	-1.299	0.003	1.058
IFNGR1	interferon gamma receptor 1	1.770	0.037	2.316	0.023	1.393	0.044	-1.253	0.021	1.057

Gene Symbol	Gene Name	Comparison #1 pSS (n=8) vs HV (n=6) Yin, H., 2013 [1]		Comparison #2 pSS (n=10) vs non-SS (n=5) Greenwell-Wild, T., 2011 [2]		Comparison #3 pSS (n=3) vs HV (n=3) Tsuboi, H., 2014 [3]		Comparison #4 pSS (n=3) vs IgG4RD (n=5) Tsuboi, H., 2014 [3]		Average of fold change (FC)
		FC	*p* value	FC	*p* value	FC	*p* value	FC	*p* value	
GTF2H3	general transcription factor IIH subunit 3	2.037	0.012	1.876	0.004	1.734	0.013	-1.434	<0.001	1.053
UBE2W	ubiquitin-conjugating enzyme E2W (putative)	1.846	0.050	1.593	0.009	2.011	0.006	-1.237	0.046	1.053
ACTR2	ARP2 actin-related protein 2 homolog (yeast)	2.304	0.016	1.840	0.002	1.500	0.024	-1.456	0.011	1.047
TNRC6B	trinucleotide repeat containing 6B	1.765	0.028	1.530	0.025	2.074	0.028	-1.194	0.048	1.043
TANK	TRAF family member-associated NFKB activator	2.014	0.043	1.726	0.033	1.711	0.024	-1.285	0.021	1.041
CTDSPL2	CTD small phosphatase like 2	1.982	0.032	1.418	0.010	1.915	0.009	-1.168	0.034	1.037
ELK3	ELK3, ETS-domain protein (SRF accessory protein 2)	1.921	0.021	1.896	0.007	1.748	0.020	-1.468	0.024	1.024
RAP2B	RAP2B, member of RAS oncogene family	2.172	0.019	1.866	0.038	1.259	0.041	-1.203	0.040	1.023
ATXN3	ataxin 3	2.518	0.012	1.618	0.004	1.405	0.044	-1.501	0.009	1.010
PARP4	poly (ADP-ribose) polymerase family member 4	2.027	0.010	1.476	0.009	1.625	0.022	-1.157	0.048	0.993
ZNF738	zinc finger protein 738	2.037	0.036	1.487	0.036	1.914	0.001	-1.526	0.002	0.978
TLR4	toll-like receptor 4	2.387	0.049	1.925	0.048	1.718	0.018	-2.269	0.001	0.940
RBPJ	recombination signal binding protein for immunoglobulin kappa J region	2.042	0.026	1.446	0.003	1.545	0.026	-1.287	0.004	0.936
ZYG11B	zyg-11 family member B, cell cycle regulator	1.697	0.045	1.527	0.013	1.691	0.043	-1.225	0.032	0.922
BNIP2	BCL2/adenovirus E1B 19kDa interacting protein 2	1.865	0.032	1.730	0.008	1.419	0.025	-1.338	0.015	0.919
FAM208A	family with sequence similarity 208, member A	1.967	0.022	1.578	0.010	1.497	0.005	-1.369	0.022	0.918
CAST	calpastatin	2.052	0.018	1.296	0.013	1.553	0.014	-1.310	0.002	0.898
LATS2	large tumor suppressor kinase 2	1.945	0.031	1.498	0.007	1.561	0.029	-1.421	0.011	0.896
PXK	PX domain containing serine/threonine kinase	1.594	0.028	1.612	0.004	1.632	0.004	-1.361	0.047	0.869
AGPAT5	1-acylglycerol-3-phosphate O-acyltransferase 5	1.637	0.045	1.849	<0.001	1.342	0.027	-1.355	0.022	0.868
STXBP3	syntaxin binding protein 3	1.881	0.031	1.527	0.028	1.368	0.046	-1.325	0.007	0.863

Gene Symbol	Gene Name	Comparison #1<br>pSS (n=8) vs HV (n=6)<br>Yin, H., 2013 [1]		Comparison #2<br>pSS (n=10) vs non-SS (n=5)<br>Greenwell-Wild, T., 2011 [2]		Comparison #3<br>pSS (n=3) vs HV (n=3)<br>Tsuboi, H., 2014 [3]		Comparison #4<br>pSS (n=3) vs IgG4RD (n=5)<br>Tsuboi, H., 2014 [3]		Average of fold change (FC)
		FC	*p* value	FC	*p* value	FC	*p* value	FC	*p* value	
ZBTB1	zinc finger and BTB domain containing 1	1.800	0.049	1.574	0.032	1.656	0.003	-1.602	0.005	0.857
G3BP1	GTPase activating protein (SH3 domain) binding protein 1	1.792	0.021	1.316	0.028	1.459	0.025	-1.159	0.031	0.852
ATE1	arginyltransferase 1	1.741	0.049	-1.198	0.026	1.602	0.030	1.261	0.009	0.852
USP1	ubiquitin specific peptidase 1	1.877	0.043	1.380	0.010	1.322	0.032	-1.219	0.044	0.840
ISCA1	iron-sulfur cluster assembly 1	1.624	0.043	1.610	0.004	1.223	0.036	-1.182	0.048	0.819
POLR1B	polymerase (RNA) I polypeptide B	1.608	0.034	1.318	0.015	1.463	0.004	-1.291	0.007	0.775
CUX1	cut-like homeobox 1	-1.933	0.024	1.809	0.003	1.375	0.008	1.222	0.009	0.618
WISP1	WNT1 inducible signaling pathway protein 1	3.791	0.025	-1.269	0.006	-1.507	0.013	1.179	0.031	0.549
NUCKS1	nuclear casein kinase and cyclin-dependent kinase substrate 1	1.812	0.013	-1.383	0.001	2.818	0.023	-1.768	0.022	0.370
CASP8	caspase 8, apoptosis-related cysteine peptidase	1.877	0.048	2.189	0.016	-1.356	0.033	-1.268	0.031	0.360
MAST4	microtubule associated serine/threonine kinase family member 4	-2.235	0.015	-1.277	0.009	2.005	0.029	2.181	0.010	0.168
VASH2	vasohibin 2	4.703	0.011	-1.397	0.009	-1.304	0.020	-1.475	0.010	0.132
CYP2U1	cytochrome P450, family 2, subfamily U, polypeptide 1	1.734	0.026	1.521	0.050	-1.267	0.033	-1.740	0.001	0.062
GM2A	GM2 ganglioside activator	-1.826	0.040	2.180	0.017	-1.387	0.019	1.204	0.040	0.043
LUZP1	leucine zipper protein 1	-1.804	0.025	1.576	0.027	1.454	0.032	-1.319	0.031	-0.023
HNRNPC	heterogeneous nuclear ribonucleoprotein C (C1/C2)	-1.737	0.026	1.300	0.008	1.381	0.049	-1.260	0.004	-0.079
SNRPN	small nuclear ribonucleoprotein polypeptide N	-2.244	0.042	1.929	0.019	1.241	0.038	-1.293	0.008	-0.092
FAM71E2	family with sequence similarity 71, member E2	3.443	0.042	-1.307	0.001	-1.341	0.014	-1.195	0.036	-0.100
CNPY3	canopy FGF signaling regulator 3	-1.747	0.016	1.449	0.011	-1.417	0.013	1.181	0.010	-0.134
CDC42EP1	CDC42 small effector 1	-2.567	0.004	-1.251	0.029	1.748	0.013	1.271	0.028	-0.200
ALAD	aminolevulinate dehydratase	-2.248	0.014	-1.463	0.003	1.591	0.049	1.299	0.011	-0.205
CALD1	caldesmon 1	-3.070	0.004	1.592	0.013	2.299	0.040	-1.853	0.001	-0.258

Gene Symbol	Gene Name	Comparison #1 pSS (n=8) vs HV (n=6) Yin, H., 2013 [1]		Comparison #2 pSS (n=10) vs non-SS (n=5) Greenwell-Wild, T., 2011 [2]		Comparison #3 pSS (n=3) vs HV (n=3) Tsuboi, H., 2014 [3]		Comparison #4 pSS (n=3) vs IgG4RD (n=5) Tsuboi, H., 2014 [3]		Average of fold change (FC)
		FC	*p* value	FC	*p* value	FC	*p* value	FC	*p* value	
RTKN	rhotekin	-2.017	0.030	-1.835	0.011	1.397	0.012	1.321	0.018	-0.284
AP2B1	adaptor-related protein complex 2, beta 1 subunit	-2.527	0.003	-2.051	0.001	1.601	0.018	1.230	0.019	-0.437
CDK19	cyclin-dependent kinase 19	1.913	0.007	-1.208	0.002	-1.455	0.004	-1.214	0.034	-0.491
PHLDA1	pleckstrin homology-like domain, family A, member 1	-3.529	0.009	-1.807	0.025	1.724	0.026	1.460	0.001	-0.538
SYNJ2	synaptojanin 2	-1.962	0.013	-1.191	0.013	1.800	0.012	-1.241	0.033	-0.648
CCNY	cyclin Y	-1.875	0.041	1.486	0.002	-1.291	0.014	-1.212	0.035	-0.723
WWTR1	WW domain containing transcription regulator 1	-1.943	0.035	-1.367	0.002	1.647	0.026	-1.323	0.013	-0.747
DCAF11	DDB1 and CUL4 associated factor 11	-1.611	0.046	-1.545	0.013	-1.264	0.016	1.409	<0.001	-0.753
SLC38A10	solute carrier family 38, member 10	-1.657	0.040	-1.727	0.003	-1.338	0.011	1.574	0.001	-0.787
BAD	BCL2-associated agonist of cell death	-1.705	0.028	-1.572	0.046	-1.341	0.013	1.229	0.032	-0.847
PXMP2	peroxisomal membrane protein 2	-1.903	0.029	-1.392	0.050	-1.419	0.038	1.317	0.008	-0.849
SARS2	seryl-tRNA synthetase 2, mitochondrial	-1.772	0.037	-1.507	0.001	-1.295	0.017	1.171	0.028	-0.851
ADCK5	aarF domain containing kinase 5	-1.809	0.041	-1.433	0.007	-1.378	0.048	1.211	0.042	-0.852
POFUT2	protein O-fucosyltransferase 2	-2.242	0.047	-1.147	0.018	-1.271	0.039	1.238	0.049	-0.856
RASAL1	RAS protein activator like 1 (GAP1 like)	-1.961	0.031	-1.332	0.021	-1.486	0.009	1.304	0.038	-0.869
SDHAF1	succinate dehydrogenase complex assembly factor 1	-1.959	0.014	-1.289	0.043	-1.477	0.009	1.214	0.008	-0.878
SPPL2B	signal peptide peptidase like 2B	-1.948	0.030	-1.444	0.001	-1.435	0.009	1.259	0.010	-0.892
MPST	mercaptopyruvate sulfurtransferase	-1.801	0.030	-1.635	0.012	-1.390	0.033	1.220	0.024	-0.901
KLC1	kinesin light chain 1	-1.985	0.013	-1.714	0.027	-1.250	0.039	1.319	0.005	-0.907
NCAPH2	non-SMC condensin II complex subunit H2	-1.882	0.036	-1.778	0.001	-1.281	0.033	1.298	0.002	-0.911
ZER1	zyg-11 related, cell cycle regulator	-1.810	0.025	-1.602	0.001	-1.754	0.047	1.401	0.001	-0.941
TRAPPC2L	trafficking protein particle complex 2-like	-2.248	0.017	-1.285	0.007	-1.524	0.008	1.193	0.038	-0.966
ZNF219	zinc finger protein 219	-2.518	0.006	-1.309	0.027	-1.368	0.004	1.253	0.047	-0.986

Gene Symbol	Gene Name	Comparison #1 pSS (n=8) vs HV (n=6) Yin, H., 2013 [1]		Comparison #2 pSS (n=10) vs non-SS (n=5) Greenwell-Wild, T., 2011 [2]		Comparison #3 pSS (n=3) vs HV (n=3) Tsuboi, H., 2014 [3]		Comparison #4 pSS (n=3) vs IgG4RD (n=5) Tsuboi, H., 2014 [3]		Average of fold change (FC)
		FC	*p* value	FC	*p* value	FC	*p* value	FC	*p* value	
CLPTM1	cleft lip and palate associated transmembrane protein 1	-2.294	0.030	-1.567	0.024	-1.343	0.014	1.224	0.022	-0.995
TMEM8A	transmembrane protein 8A	-1.756	0.050	-2.195	0.002	-1.319	0.019	1.283	0.004	-0.997
SCRN2	secernin 2	-2.187	0.020	-1.572	0.001	-1.428	0.029	1.182	0.020	-1.001
STUB1	STIP1 homology and U-box containing protein 1, E3 ubiquitin protein ligase	-2.093	0.015	-1.796	0.006	-1.325	0.029	1.193	0.023	-1.005
GHDC	GH3 domain containing	-2.027	0.023	-2.022	0.003	-1.290	0.031	1.306	0.023	-1.008
FBXL15	F-box and leucine-rich repeat protein 15	-1.639	0.039	-1.930	0.005	-1.657	0.005	1.192	0.034	-1.009
NEDD4L	neural precursor cell expressed, developmentally down-regulated 4-like, E3 ubiquitin protein ligase	-1.787	0.047	-2.203	0.002	1.365	0.013	-1.424	0.025	-1.012
PGLS	6-phosphogluconolactonase	-1.838	0.021	-1.988	0.024	-1.441	0.024	1.197	0.026	-1.017
CDH23	cadherin-related 23	-2.633	0.023	-1.266	0.024	-1.420	0.008	1.244	0.017	-1.019
GPS1	G protein pathway suppressor 1	-2.141	0.007	-1.889	0.007	-1.307	0.038	1.255	0.020	-1.021
TMEM132A	transmembrane protein 132A	-2.499	0.033	-1.318	0.009	-1.452	0.024	1.160	0.041	-1.027
TBC1D16	TBC1 domain family, member 16	-2.426	0.038	-1.765	0.001	-1.244	0.044	1.301	0.033	-1.033
SCAND1	SCAN domain containing 1	-2.094	0.042	-1.911	0.004	-1.318	0.043	1.164	0.048	-1.040
HM13	histocompatibility (minor) 13	-1.731	0.035	-2.359	0.007	-1.316	0.045	1.234	0.025	-1.043
AUP1	ancient ubiquitous protein 1	-2.214	0.023	-1.699	0.006	-1.456	0.007	1.162	0.025	-1.052
SGSM3	small G protein signaling modulator 3	-2.321	0.036	-2.023	0.004	-1.238	0.046	1.374	0.015	-1.052
LMF1	lipase maturation factor 1	-1.877	0.034	-2.485	0.001	-1.342	0.025	1.463	0.003	-1.060
SLC25A29	solute carrier family 25 (mitochondrial carnitine/acylcarnitine carrier), member 29	-2.426	0.024	-1.749	0.001	-1.276	0.047	1.183	0.043	-1.067
CORO1B	coronin, actin binding protein, 1B	-2.326	0.006	-2.044	0.001	-1.334	0.017	1.431	0.002	-1.068
CERCAM	cerebral endothelial cell adhesion molecule	-1.948	0.033	-2.017	<0.001	-1.591	0.005	1.273	0.001	-1.071
PHLDB3	pleckstrin homology-like domain, family B, member 3	-1.982	0.011	-2.413	0.001	-1.339	0.033	1.365	0.002	-1.092
COPE	coatomer protein complex subunit epsilon	-2.525	0.012	-1.665	0.026	-1.425	0.009	1.236	0.030	-1.095

Gene Symbol	Gene Name	Comparison #1 pSS (n=8) vs HV (n=6) Yin, H., 2013 [1]		Comparison #2 pSS (n=10) vs non-SS (n=5) Greenwell-Wild, T., 2011 [2]		Comparison #3 pSS (n=3) vs HV (n=3) Tsuboi, H., 2014 [3]		Comparison #4 pSS (n=3) vs IgG4RD (n=5) Tsuboi, H., 2014 [3]		Average of fold change (FC)
		FC	*p* value	FC	*p* value	FC	*p* value	FC	*p* value	
MAGIX	MAGI family member, X-linked	-2.462	0.040	-1.923	<0.001	-1.408	0.014	1.407	0.026	-1.096
RASSF7	Ras association (RalGDS/AF-6) domain family (N-terminal) member 7	-2.598	0.018	-1.644	0.001	-1.606	0.010	1.364	0.001	-1.121
ATP5D	ATP synthase, H+ transporting, mitochondrial F1 complex, delta subunit	-1.914	0.028	-2.465	0.001	-1.316	0.035	1.187	0.038	-1.127
TEX264	testis expressed 264	-2.018	0.016	-2.162	0.002	-1.564	0.009	1.233	0.022	-1.128
ZDHHC12	zinc finger, DHHC-type containing 12	-2.322	0.015	-1.859	0.002	-1.520	0.028	1.188	0.038	-1.128
SERINC2	serine incorporator 2	-2.523	0.015	-1.947	0.009	-1.389	0.026	1.310	0.012	-1.137
ACAP3	ArfGAP with coiled-coil, ankyrin repeat and PH domains 3	-2.637	0.027	-1.840	0.003	-1.489	0.013	1.316	0.004	-1.163
MXD4	MAX dimerization protein 4	-2.677	0.009	-1.918	<0.001	-1.296	0.036	1.234	0.008	-1.164
SLC13A2	solute carrier family 13 (sodium-dependent dicarboxylate transporter), member 2	-2.103	0.010	-2.546	<0.001	-1.492	0.027	1.464	0.005	-1.169
B4GALNT3	beta-1,4-N-acetyl-galactosaminyl transferase 3	-3.066	0.013	-1.414	0.013	-1.485	0.007	1.190	0.013	-1.193
BAIAP2	BAI1-associated protein 2	-2.271	0.003	-2.565	0.002	-1.338	0.044	1.391	0.018	-1.196
SOX2	SRY box 2	-2.228	0.047	-2.467	<0.001	-1.349	0.050	1.237	0.011	-1.202
S100A1	S100 calcium binding protein A1	-3.042	0.035	-1.745	0.002	-1.341	0.035	1.290	0.007	-1.209
KLHDC3	kelch domain containing 3	-2.251	0.031	-2.416	0.001	-1.434	0.046	1.242	0.008	-1.215
CERS4	ceramide synthase 4	-2.205	0.009	-2.436	0.001	-1.570	0.006	1.349	0.017	-1.215
SETD5	SET domain containing 5	-3.217	0.004	-1.416	0.003	-1.656	0.020	1.303	0.039	-1.246
AGFG2	ArfGAP with FG repeats 2	-3.049	0.028	-2.009	0.001	-1.277	0.042	1.330	0.030	-1.251
TM7SF2	transmembrane 7 superfamily member 2	-2.710	0.031	-2.225	0.001	-1.607	0.025	1.464	0.008	-1.270
LRP5	LDL receptor related protein 5	-2.714	0.011	-2.279	0.008	-1.389	0.040	1.274	0.027	-1.277
ADAM15	ADAM metallopeptidase domain 15	-3.465	0.002	-1.365	0.045	-1.590	0.018	1.310	0.018	-1.278
SEZ6L2	seizure related 6 homolog (mouse)-like 2	-2.836	0.028	-2.271	0.001	-1.638	0.013	1.584	0.010	-1.290
SOX4	SRY box 4	-2.904	0.009	-1.894	0.025	-1.600	0.009	1.172	0.049	-1.306
EDA	ectodysplasin A	-1.543	0.045	-1.340	0.013	-1.262	0.027	-1.209	0.036	-1.338

Gene Symbol	Gene Name	Comparison #1 pSS (n=8) vs HV (n=6) Yin, H., 2013 [1]		Comparison #2 pSS (n=10) vs non-SS (n=5) Greenwell-Wild, T., 2011 [2]		Comparison #3 pSS (n=3) vs HV (n=3) Tsuboi, H., 2014 [3]		Comparison #4 pSS (n=3) vs IgG4RD (n=5) Tsuboi, H., 2014 [3]		Average of fold change (FC)
		FC	*p* value	FC	*p* value	FC	*p* value	FC	*p* value	
DNAJC4	DnaJ (Hsp40) homolog, subfamily C, member 4	-2.553	0.004	-2.690	0.002	-1.451	0.011	1.263	0.007	-1.358
RIMS3	regulating synaptic membrane exocytosis 3	-1.780	0.030	-1.174	0.033	-1.371	0.012	-1.398	0.009	-1.431
WASF2	WAS protein family, member 2	-3.585	0.010	-2.103	0.002	1.388	0.011	-1.578	0.009	-1.469
PITX1	paired-like homeodomain 1	-3.095	0.018	-2.771	0.001	-1.415	0.012	1.390	0.015	-1.473
TNS1	tensin 1	-1.810	0.038	-1.400	0.022	-1.211	0.047	-1.547	0.031	-1.492
SMARCD3	SWI/SNF related, matrix associated, actin dependent regulator of chromatin, subfamily d, member 3	-1.700	0.046	-1.592	0.008	-1.305	0.045	-1.374	0.039	-1.493
STARD10	StAR-related lipid transfer domain containing 10	-2.343	0.015	-3.579	0.004	-1.546	0.017	1.433	0.004	-1.509
NAV2	neuron navigator 2	-2.011	0.038	-1.228	0.036	-1.274	0.027	-1.545	0.015	-1.514
GATA2	GATA binding protein 2	-1.800	0.049	-1.296	0.028	-1.631	0.005	-1.453	0.015	-1.545
NADK	NAD kinase	-4.451	0.004	-1.727	0.001	-1.347	0.011	1.326	0.041	-1.550
SCD5	stearoyl-CoA desaturase 5	-1.971	0.025	-1.226	0.021	-1.354	0.014	-1.678	0.009	-1.557
GPSM1	G-protein signaling modulator 1	-1.965	0.027	-1.503	<0.001	-1.420	0.046	-1.372	0.026	-1.565
ZC3H7B	zinc finger CCCH-type containing 7B	-2.202	0.021	-1.200	0.033	-1.266	0.021	-1.653	0.001	-1.580
FOSL2	FOS-like antigen 2	-2.120	0.020	-1.507	0.010	-1.430	0.020	-1.317	0.046	-1.594
ECE1	endothelin converting enzyme 1	-2.575	0.038	-1.274	0.026	-1.303	0.015	-1.263	0.010	-1.604
BTNL9	butyrophilin-like 9	-1.933	0.031	-1.217	0.047	-1.393	0.014	-1.879	0.017	-1.606
WASH1	WAS protein family homolog 1	-2.236	0.015	-1.371	0.018	-1.306	0.040	-1.532	0.007	-1.611
JPH2	junctophilin 2	-2.197	0.038	-1.356	0.027	-1.494	0.018	-1.476	0.037	-1.631
RECQL5	RecQ helicase-like 5	-2.340	0.012	-1.235	0.039	-1.468	0.016	-1.498	<0.001	-1.635
THRA	thyroid hormone receptor, alpha	-2.323	0.021	-1.603	0.001	-1.663	0.031	-1.231	0.045	-1.705
BOC	BOC cell adhesion associated, oncogene regulated	-2.393	0.047	-1.557	0.001	-1.335	0.010	-1.550	0.018	-1.709
UBQLN4	ubiquilin 4	-2.568	0.005	-1.390	0.002	-1.500	0.024	-1.510	<0.001	-1.742
ACTN1	actinin, alpha 1	-2.300	0.018	-2.172	<0.001	-1.283	0.020	-1.226	0.023	-1.745
DBP	D site of albumin promoter (albumin D-box) binding protein	-2.253	0.028	-2.366	0.015	-1.400	0.020	-1.218	0.019	-1.809

Gene Symbol	Gene Name	Comparison #1 pSS (n=8) vs HV (n=6) Yin, H., 2013 [1]		Comparison #2 pSS (n=10) vs non-SS (n=5) Greenwell-Wild, T., 2011 [2]		Comparison #3 pSS (n=3) vs HV (n=3) Tsuboi, H., 2014 [3]		Comparison #4 pSS (n=3) vs IgG4RD (n=5) Tsuboi, H., 2014 [3]		Average of fold change (FC)
		FC	*p* value	FC	*p* value	FC	*p* value	FC	*p* value	
LZTS2	leucine zipper, putative tumor suppressor 2	-3.107	0.039	-1.641	0.034	-1.331	0.031	-1.234	0.029	-1.828
PBX2	pre-B-cell leukemia homeobox 2	-3.329	<0.001	-1.494	0.004	-1.250	0.045	-1.302	0.047	-1.844
KLK3	kallikrein related peptidase 3	-3.735	0.001	-1.240	0.004	-1.452	0.035	-1.300	0.043	-1.932
CAPS	calcyphosine	-2.535	0.023	-2.152	0.011	-1.807	0.001	-1.415	0.024	-1.977
TPM2	tropomyosin 2 (beta)	-3.531	0.024	-1.240	0.025	-1.364	0.030	-1.932	0.003	-2.017
TAGLN	transgelin	-3.307	0.022	-2.195	0.018	-1.477	0.004	-1.510	0.044	-2.122
KRT14	keratin 14, type I	-3.467	0.032	-1.494	0.033	-1.803	0.006	-1.750	0.041	-2.128
METRN	meteorin, glial cell differentiation regulator	-5.674	0.001	-2.506	0.001	-1.670	0.003	1.284	0.033	-2.141
AKT2	v-akt murine thymoma viral oncogene homolog 2	-4.306	0.007	-1.953	0.004	-1.370	0.017	-1.299	0.022	-2.232
KRT17	keratin 17, type I	-3.392	0.038	-2.646	0.026	-1.846	0.004	-1.673	0.030	-2.389
VSIG2	V-set and immunoglobulin domain containing 2	-3.346	0.001	-2.397	0.002	-2.753	0.028	-2.151	0.021	-2.662

* Fold change (FC) and p values were calculated by GeneSpring Multi-Omic Analysis (version 14.9-Build 11939, Agilent Technologies, Inc.), using Moderated T-Test with asymptotic p-value computation and Benjamini Hochberg FDR for multiple testing correlation.
pSS: primary Sjögren syndrome; HV: healthy volunteers; IgG4RS: IgG4 related diseases

Reference

1. Yin, H. *et al.* Association of Bone Morphogenetic Protein 6 With Exocrine Gland Dysfunction in Patients With Sjögren's Syndrome and in Mice. *Arthritis Rheum.* **65**(12): 3228–3238 (2013).
2. Greenwell-Wild, T. *et al.* Chitinases in the salivary glands and circulation of patients with Sjögren's syndrome: macrophage harbingers of disease severity. *Arthritis Rheum.* **63**(10): 3103-15 (2011).
3. Tsuboi, H. *et al.* DNA microarray analysis of labial salivary glands in IgG4-related disease: comparison with Sjögren's syndrome. *Arthritis Rheumatol.* **66**(10):2892-9 (2014).

Supplementary Table S2. Common canonical pathways in minor salivary glands among four comparisons from 3 transcriptome studies

Canonical Pathways	Comparison #1<br>pSS (n=8) vs HV (n=6)<br>Yin, H., 2013 [1]	Comparison #2<br>pSS (n=10) vs non-SS (n=5)<br>Greenwell-Wild, T., 2011 [2]	Comparison #3<br>pSS (n=3) vs HV (n=3)<br>Tsuboi, H., 2014 [3]	Comparison #4<br>pSS (n=3) vs IgG4RD (n=5)<br>Tsuboi, H., 2014 [3]
Interferon Signaling	2.138***	2.183**	2.183**	2.357***
PKCθ Signaling in T Lymphocytes	3.087***	0.535**	4.628***	0.164*
Role of Pattern Recognition Receptors in Recognition of Bacteria and Viruses	3.266**	0.905**	2.287*	0.539**
RhoGDI Signaling	2.058***	1.941*	1.860***	0.469**
ERK5 Signaling	0.426***	2.828**	1.761***	0.816**
Activation of IRF by Cytosolic Pattern Recognition Receptors	2.138*	0.822***	1.512*	1.342*
SPINK1 General Cancer Pathway	0.655***	1.718***	2.214**	1.095***
FcγRIIB Signaling in B Lymphocytes	2.000*	1.095***	2.335*	0.229**
B Cell Receptor Signaling	1.820***	0.556***	2.800***	0.412***
Leukocyte Extravasation Signaling	2.412***	0.611*	1.254**	0.149*
PI3K Signaling in B Lymphocytes	1.000***	0.739***	2.077***	0.343*
IL-9 Signaling	2.121*	0.655*	0.218*	0.471**
Th1 Pathway	4.017***	1.333	2.111	0.354*
Retinoic acid Mediated Apoptosis Signaling	2.309*	0.600*	1.961*	0.905
Role of BRCA1 in DNA Damage Response	1.414	2.400***	1.213*	0.378
Prolactin Signaling	1.877***	0.457***	1.286***	0.928**
SAPK/JNK Signaling	1.460**	0.429***	1.987***	0.632***
Calcium-induced T Lymphocyte Apoptosis	1.387*	0.688	1.671**	0.277
Tec Kinase Signaling	1.333***	0.405**	1.778***	0.160**
Apelin Endothelial Signaling Pathway	1.183***	1.033***	1.543***	0.632**
Aldosterone Signaling in Epithelial Cells	0.853***	0.316**	0.762***	0.186***
UVA-Induced MAPK Signaling	0.853***	0.649***	1.372***	0.209***
Gα12/13 Signaling	0.667***	0.130***	0.843***	0.152**
ErbB4 Signaling	0.655***	0.343**	1.761***	0.200***
Actin Nucleation by ARP-WASP Complex	0.655***	0.200**	1.151***	1.291
CNTF Signaling	0.626***	0.845***	1.48***	0.655*
IL-3 Signaling	0.600***	0.905***	1.286***	0.522***
Endothelin-1 Signaling	0.590***	0.333***	1.746**	0.911***

Canonical Pathways	Comparison #1<br>pSS (n=8) vs HV (n=6)<br>Yin, H., 2013 [1]	Comparison #2<br>pSS (n=10) vs non-SS (n=5)<br>Greenwell-Wild, T., 2011 [2]	Comparison #3<br>pSS (n=3) vs HV (n=3)<br>Tsuboi, H., 2014 [3]	Comparison #4<br>pSS (n=3) vs IgG4RD (n=5)<br>Tsuboi, H., 2014 [3]
PDGF Signaling	0.557***	1.021***	0.980***	0.686**
p53 Signaling	0.447***	0.745***	0.420***	0.557***
Insulin Receptor Signaling	0.343***	0.662**	0.894***	0.457**
FLT3 Signaling in Hematopoietic Progenitor Cells	0.186***	0.745***	1.540***	0.174***
Role of RIG1-like Receptors in Antiviral Innate Immunity	1.414	0.229**	0.832	0.905
Small Cell Lung Cancer Signaling	-0.243**	-0.686***	-0.324***	-0.655*
Bladder Cancer Signaling	-0.577**	-1.414*	-0.688**	-1.265
Apelin Adipocyte Signaling Pathway	-0.577	-0.816	-1.298**	-1.147
Apelin Cardiomyocyte Signaling Pathway	-0.756***	-1.089***	-0.980***	-1.616*
Basal Cell Carcinoma Signaling	-1	-1.460**	-1.460*	-1.069
cAMP-mediated signaling	-1.3	-0.459**	-0.113*	-1.131*
ILK Signaling	-1.769***	-0.459***	-0.954***	-1.807***
Adrenomedullin signaling pathway	-1.570***	-0.110***	-1.043***	-0.250***
Neuroprotective Role of THOP1 in Alzheimer's Disease	-1.732	-3.674	-2.667*	-0.229
GPCR-Mediated Nutrient Sensing in Enteroendocrine Cells	-0.626*	-2.236*	-1.093	-1.826
Calcium Signaling	-0.894	-3.200***	-0.832*	-1.372*
Amyloid Processing	-1.414**	-1.897*	-2.183***	-1.667*

Data are ranked based on Z scores of the pathways (statistical measure of the match between expected relationship direction and observed gene expression) calculated by Ingenuity Pathway Analysis software. Red indicates positive Z scores, which predicted activation, and dark red indicated Z scores >2. Blue indicates a negative Z scores, which predicts inhibition, and dark blue indicates Z scores <-2. P values of Z scores were calculated using Fisher's exact test.

**p*<0.05; ***p*<0.01; ****p*<0.001

pSS: primary Sjögren syndrome; HV: healthy volunteers; IgG4RS: IgG4 related diseases

Reference

1. Yin, H. *et al.* Association of Bone Morphogenetic Protein 6 With Exocrine Gland Dysfunction in Patients With Sjögren’s Syndrome and in Mice. *Arthritis Rheum.* **65**(12): 3228–3238 (2013).
2. Greenwell-Wild, T. *et al.* Chitinases in the salivary glands and circulation of patients with Sjögren's syndrome: macrophage harbingers of disease severity. *Arthritis Rheum.* **63**(10): 3103-15 (2011).
3. Tsuboi, H. *et al*. DNA microarray analysis of labial salivary glands in IgG4-related disease: comparison with Sjögren's syndrome. *Arthritis Rheumatol.* **66**(10):2892-9 (2014).

Supplementary Table S3

Comparison #1 pSS (n=8) vs HV (n=6) (Yin H, 2013 [1])

Subject no.	Group	Gender	Age
pSS-37	pSS	F	62
pSS-94	pSS	F	60
pSS-101	pSS	F	58
pSS-105	pSS	F	42
pSS-106	pSS	F	31
pSS-139	pSS	F	46
pSS-141	pSS	F	55
pSS-144	pSS	F	50
HV-81	HV	F	54
HV-85	HV	F	37
HV-86	HV	F	53
HV-88	HV	F	34
HV-102	HV	F	44
HV-122	HV	F	30

Comparison #2: pSS (n=10) vs non-SS (n=5) (Greenwell-Wild T, 2011 [2])

Subject no.	Group	Gender	Age
C1	Non-SS (PBC; allergies)	F	53
C2	Non-SS (Psoriatic arthritis)	F	51
C3	Non-SS (arthralgia, myalgia, muscle weakness, scleroderma)	F	54
C4	Non-SS (dry tongue, tuberculosis)	F	43
C5	Non-SS (SLE)	F	30
E1	pSS	F	60
E2	pSS	F	41
E3	pSS	F	47
E4	pSS	F	27
E5	pSS	F	50
I1	pSS	F	54
I2	pSS	F	51
I3	pSS	F	63
S1	pSS	F	54
S2	pSS	F	32

Comparison #3 & #4 (Tsuboi H, 2014 [3])

Subject no.*	Group	Gender	Age
SS1	pSS	F	22
SS2	pSS	F	37
SS5	pSS	F	36
HV1	HV	F	65
HV2	HV	F	38
HV3	HV	F	23
IgG4RD1	IgG4RD	F	57
IgG4RD2	IgG4RD	F	61
IgG4RD3	IgG4RD	F	56
IgG4RD4	IgG4RD	F	58
IgG4RD5	IgG4RD	F	61

Reference

1. Yin, H. *et al*. Association of Bone Morphogenetic Protein 6 With Exocrine Gland Dysfunction in Patients With Sjögren’s Syndrome and in Mice. *Arthritis Rheum.* **65**(12): 3228–3238 (2013).
2. Greenwell-Wild, T. *et al*. Chitinases in the salivary glands and circulation of patients with Sjögren's syndrome: macrophage harbingers of disease severity. *Arthritis Rheum.* **63**(10): 3103-15 (2011).
3. Tsuboi, H. *et al*. DNA microarray analysis of labial salivary glands in IgG4-related disease: comparison with Sjögren's syndrome. *Arthritis Rheumatol.* **66**(10):2892-9 (2014).

Supplementary Table S4. Clinical feature of pSS patients and HV used to establish primary cells

pSGECs	Sex	SSA	SSB	FS	WUS <1.5 mL/15 min
HV_#3254	F	No	No	0	No
HV_#3267	F	No	No	0	No
HV_#3274	M	No	No	0	No
HV_#3214	F	No	No	2	No
HV_#3204	F	No	No	0	Yes
pSS_#2935	F	No	Yes	1	Yes
pSS_#3098	F	Yes	No	2	Yes
pSS_#3013	F	Yes	Yes	2	Yes
pSS_#3237	F	Yes	No	1	Yes
pSS_#3247	F	Yes	No	1	No
pSS_#3371	M	Yes	Yes	1	No
pSS_#3485	M	Yes	Yes	3	Yes
pSS_#3203	F	No	No	1	Yes

Supplementary Figure S1

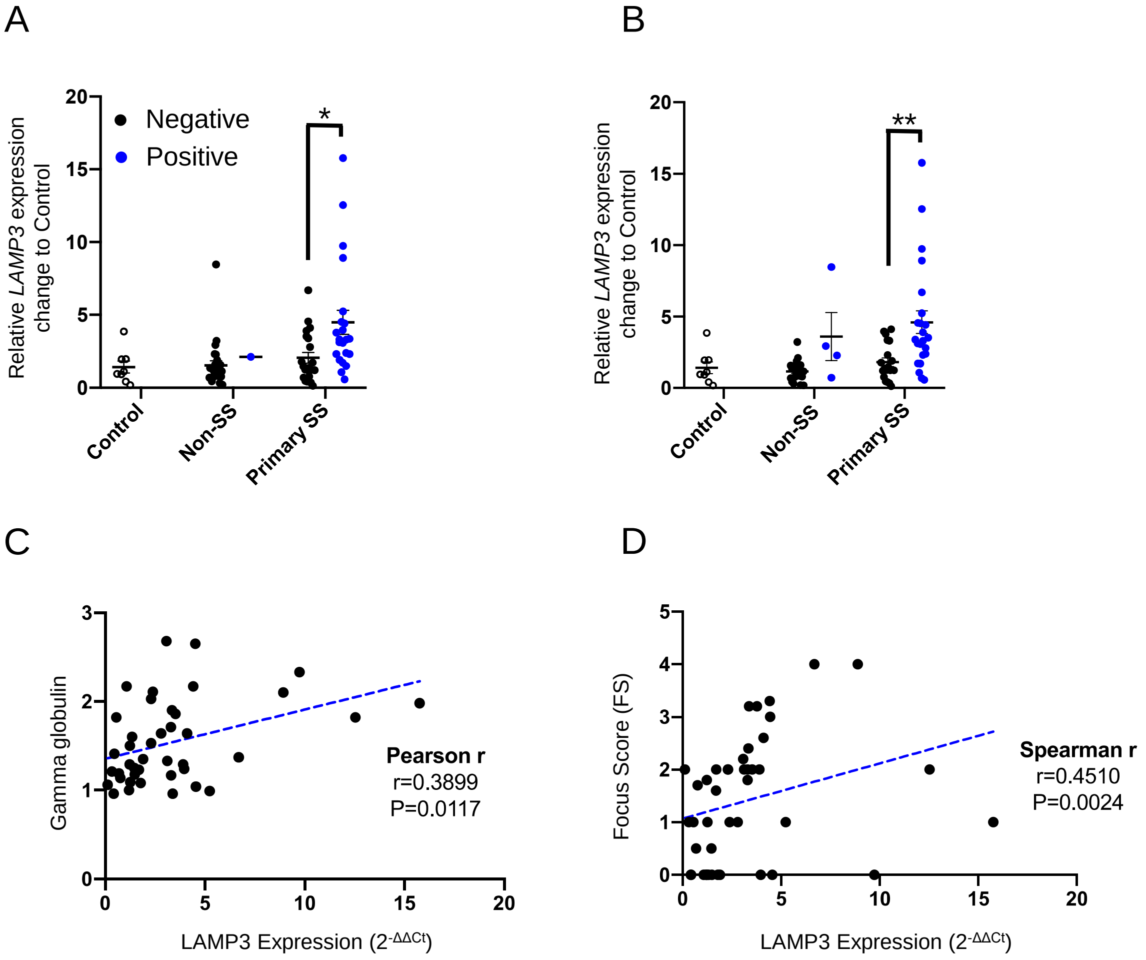

Supplementary Figure S1.

LAMP3 correlates with autoantibodies and gamma globulin levels in pSS patients. (A, B) Patients were divided based on clinical presentation of autoantibodies (**A.** anti La/SSB, **B.** ANA) and then data plotted vs LAMP3 mRNA expression observed in non-SS and pSS. **(C)** Patients serum level of gamma globulin as measured by Serum Protein Electrophoresis (SPE) was compared with LAMP3 mRNA expression levels. The present of gamma globulin within the range (0.6-1.79g/dL) was defined as normal. **(D)** Patients focus score was calculated following ACR 2016 criteria (foci/4 mm^2) and compared with LAMP3 mRNA expression. *p<0.05, **p<0.01, unpaired Student's *t*-test.

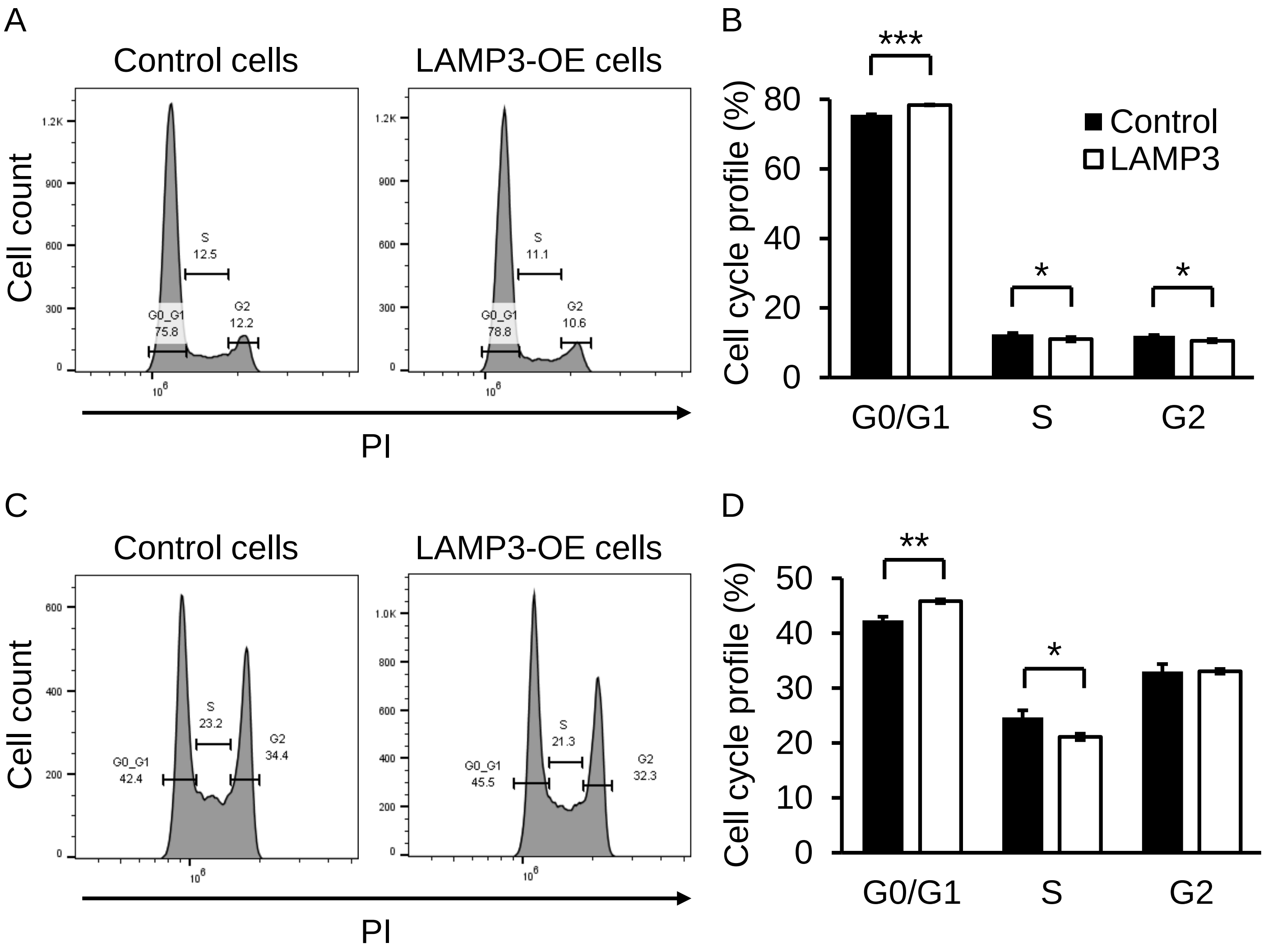

Supplementary Figure S2.

LAMP3 induced G0/G1 arrest. Cell cycle analysis was performed in controls and LAMP3-OE cells of (**A, B**) HSG and (**C, D**) A253 cells. * p<0.05, ** p<0.01, *** p<0.001, unpaired Student's t-test.

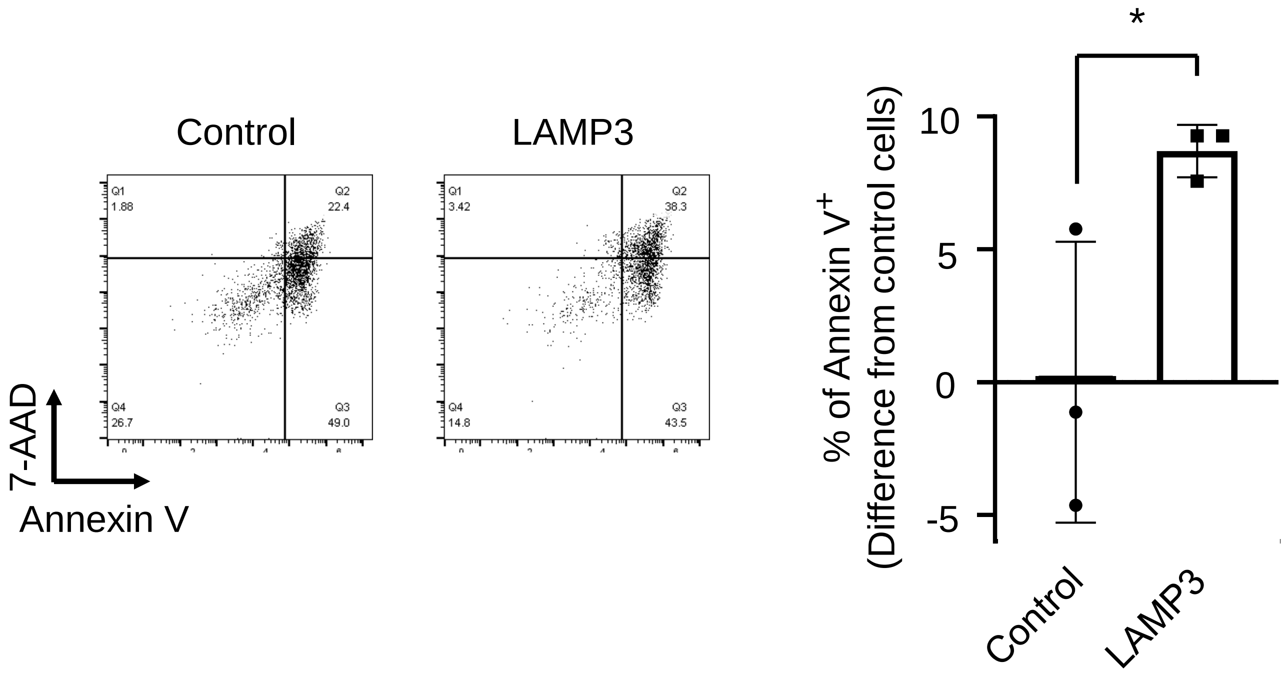

Supplementary Figure S3.

LAMP3 expression in NS-SV-AC cells induced apoptosis. NS-SV-AC cells were transfected with pME18S-LAMP3 plasmid or a pME18S-empty plasmid as control. Forty-eight hours post-transfection, apoptotic cells were counted by using flow cytometry with APC Annexin V/7-AAD. Difference from control cells is shown (N = 3). * $p<0.05$, unpaired Student's *t*-test.

Supplementary Figure S4

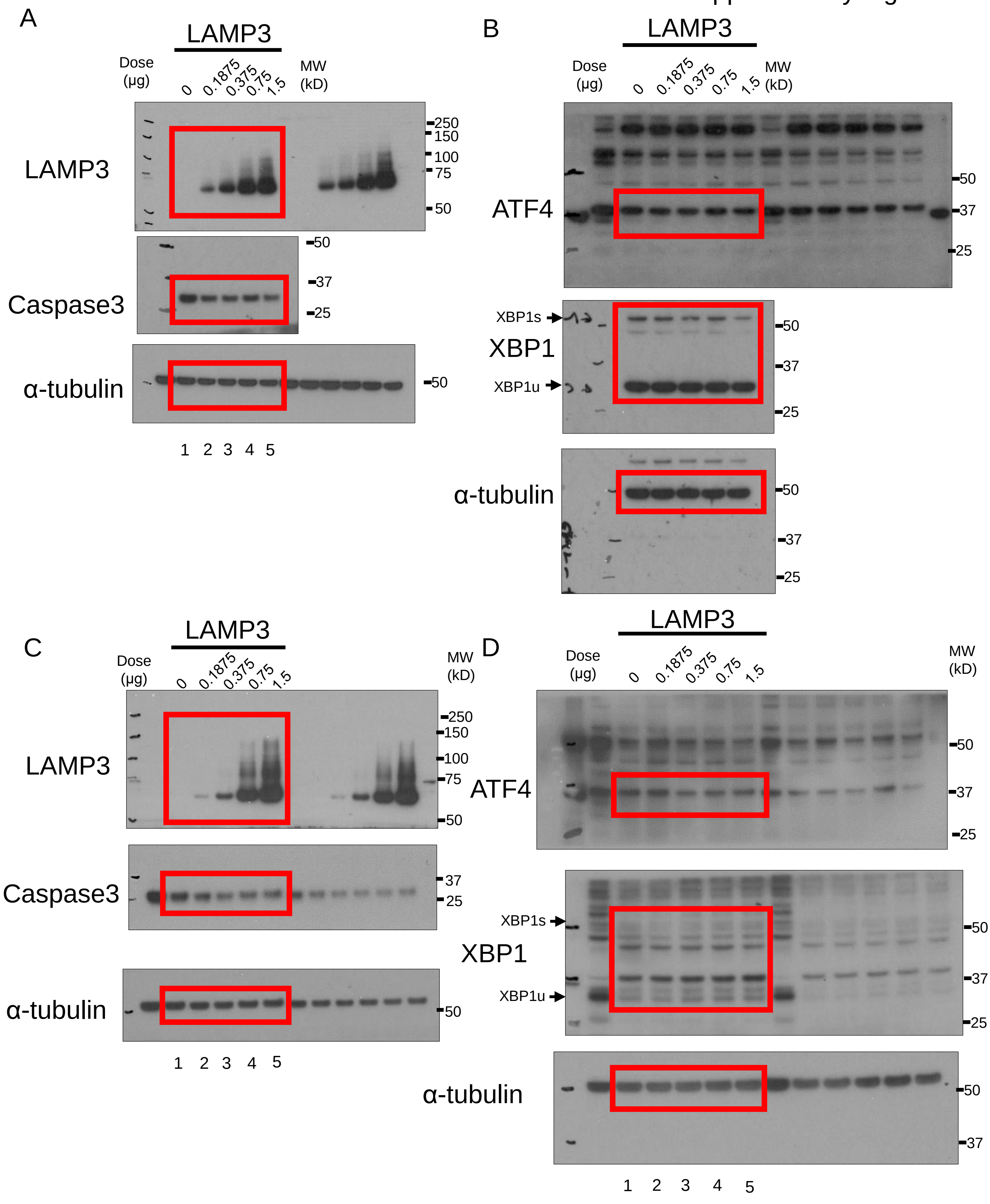

(continue to next page)

Supplementary Figure S4. Full, uncropped images of western blot membrane with molecular weight (MW) ladder from Figure 4 in which each result is boxed in red. **(A)** Full, uncropped images of western blot membrane from Figure 4B. **(B)** Full, uncropped images of western blot membrane from Figure 4C. **(C)** Full, uncropped images of western blot membrane from Figure 4E. **(D)** Full, uncropped images of western blot membrane from Figure 4F.

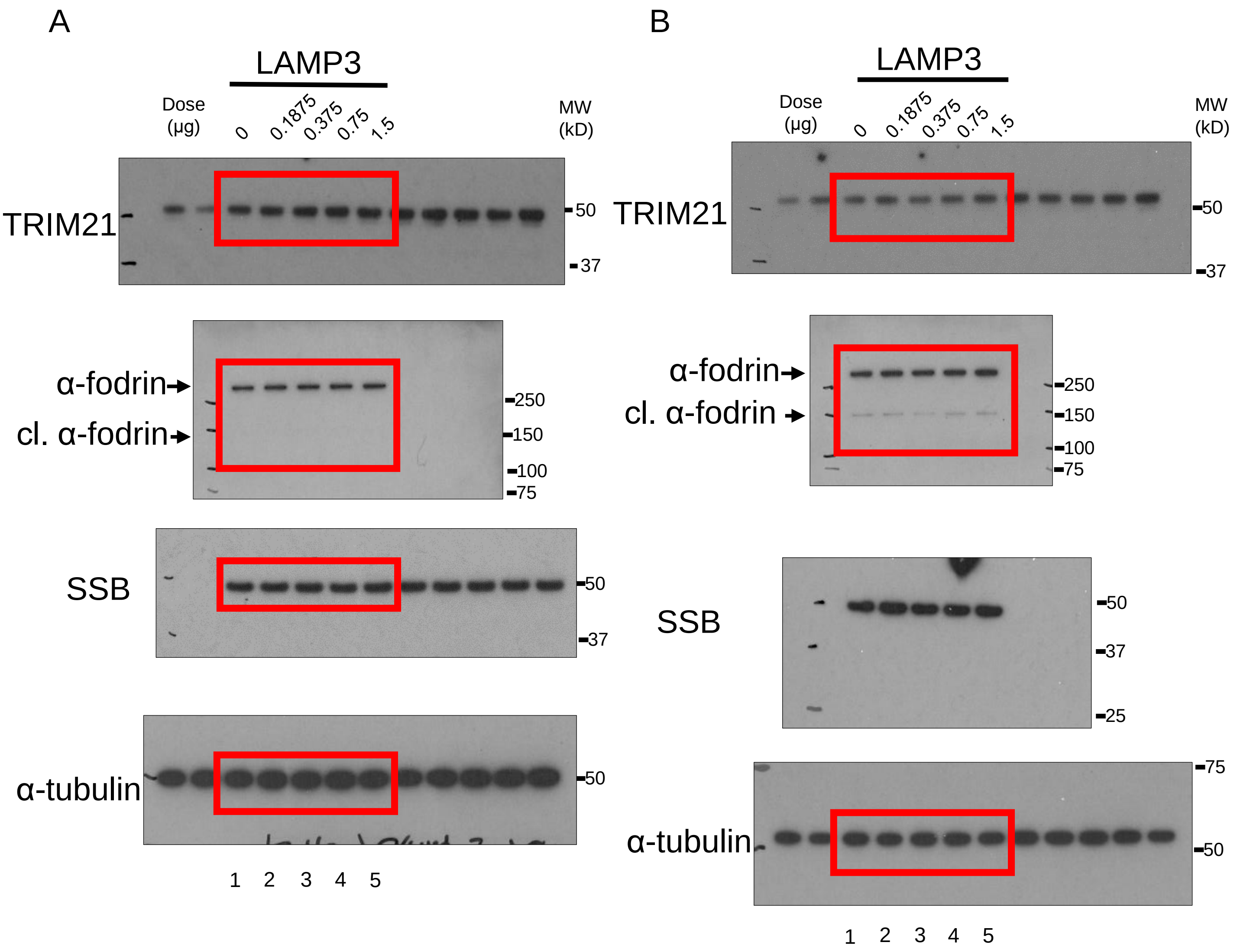

(continue to next page)

Supplementary Figure S5 (continued)

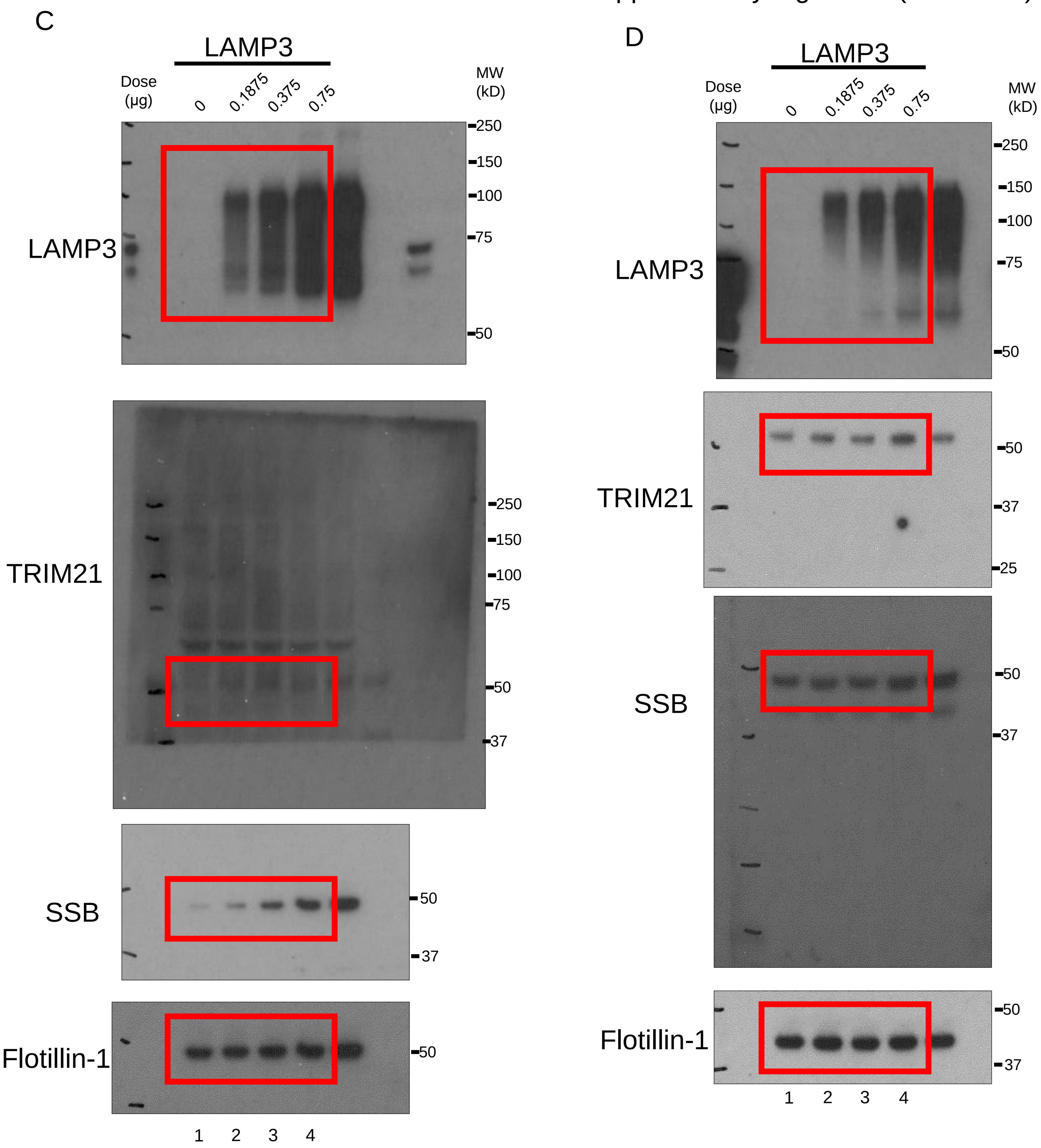

(continue to next page)

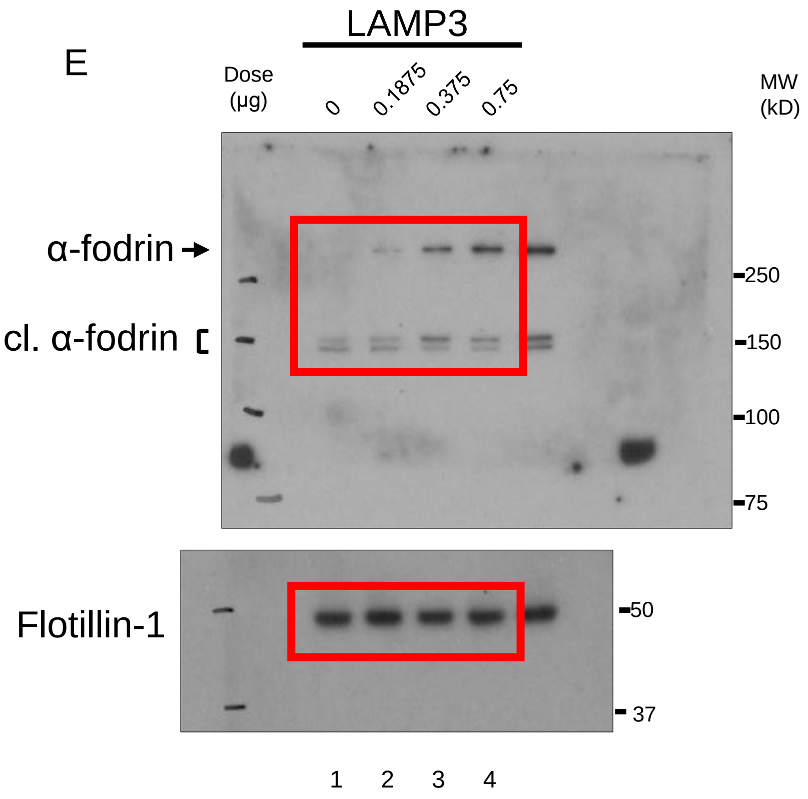

Supplementary Figure S5. Full, uncropped images of western blot membrane with molecular weight (MW) ladder from Figure 5 in which each result is boxed in red. **(A)** Full, uncropped images of western blot membrane from Figure 5B. **(B)** Full, uncropped images of western blot membrane from Figure 5C. **(C)** Full, uncropped images of western blot membrane from Figure 5E. **(D)** Full, uncropped images of western blot membrane from Figure 5F. **(E)** Full, uncropped images of western blot membrane from Figure 5G.

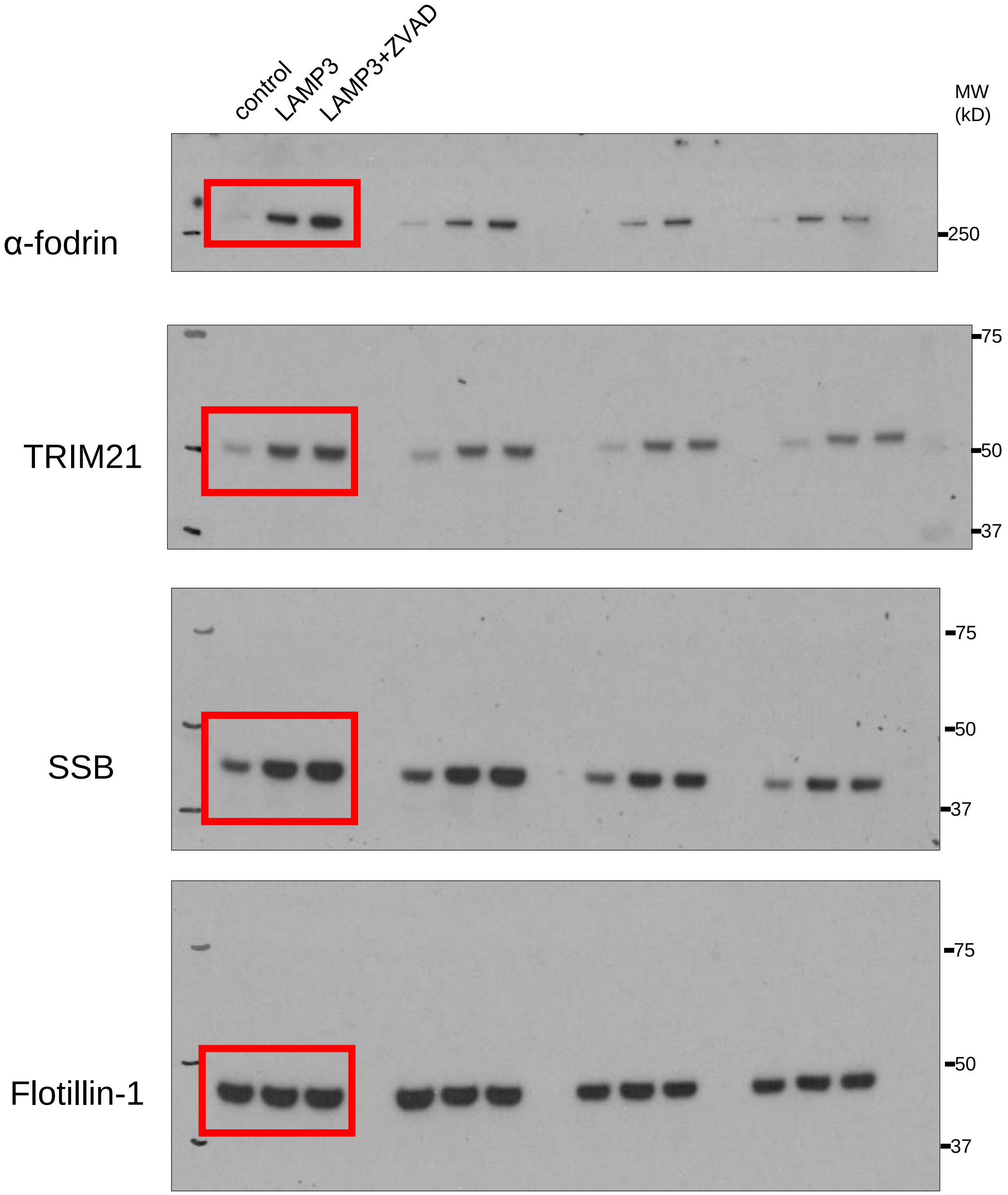

Supplementary Figure S6. Full, uncropped images of western blot membrane with molecular weight (MW) ladder from Figure 6B in which each result is boxed in red.
